# A Comprehensive Transcriptome Atlas Reveals the Crucial Role of LncRNAs in Maintaining Nodulation Homeostasis in Soybean

**DOI:** 10.1002/advs.202412104

**Published:** 2024-12-24

**Authors:** Yanru Lin, Chong Chen, Weizhen Chen, Hangcheng Liu, Renhao Xiao, Hongtao Ji, Xia Li

**Affiliations:** ^1^ National Key Laboratory of Crop Genetic Improvement Hubei Hongshan Laboratory College of Plant Science and Technology Huazhong Agricultural University Wuhan 430070 P. R. China

**Keywords:** ceRNA, lncRNA, ncRNA, nodulation, soybean

## Abstract

Symbiotic nitrogen fixation (SNF) provides nitrogen for soybean. A primary challenge in enhancing yield through efficient SNF lies in striking a balance between its high energy consumption and plant growth. However, the systemic transcriptional reprogramming during nodulation remains limited. Here, this work conducts a comprehensive RNA‐seq of the roots, cotyledons and leaves of inoculated‐soybean. This work finds 88,814 mRNAs and 6,156 noncoding RNAs (ncRNAs) across various organs. Notably, this work identifies 6,679 nodulation‐regulated mRNAs (NR‐mRNAs), 1,681 long noncoding RNAs (lncRNAs) (NR‐lncRNAs), and 59 miRNAs (NR‐miRNAs). The majority of these NR‐RNAs are associated with plant‐microbial interaction and exhibit high organ specificity. Roots display the highest abundance of NR‐ncRNAs and the most dynamic crosstalk between NR‐lncRNAs and NR‐miRNAs in a GmNARK‐dependent manner. This indicates that while each tissue responds uniquely, GmNARK serves as a primary regulator of the transcriptional control of nodulated‐plants. Furthermore, this work proves that lnc‐NNR6788 and lnc‐NNR7059 promote nodulation by regulating their target genes. This work also shows that the nodulation‐ and GmNARK‐regulated (NNR) lnc‐NNR4481 negatively regulates nodulation through miR172c within a competing endogenous RNA (ceRNA) network. The spatial organ‐type transcriptomic atlas establishes a benchmark and provides a valuable resource for integrative analyses of the mechanism underlying of nodulation and plant growth balance.

## Graphical abstract

Symbiotic nitrogen fixation promotes soybean growth, but maintaining energy balance is challenging. RNA‐seq of three organs of inoculated soybean identifies 88,814 mRNAs and 6,156 ncRNAs. Roots exhibit the most NR‐ncRNAs and dynamic crosstalk controlled by GmNARK. Lnc‐NNR6788 and lnc‐NNR7059 boost nodulation, whereas lnc‐NNR4481 inhibits it in a ceRNA network. This atlas facilitates the understanding of balancing nodulation and growth.

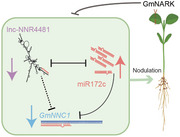

## 1 Introduction

Symbiotic nitrogen fixation (SNF) is a superior trait that enables soybean plants to acquire nearly sufficient nitrogen to ensure ideal growth and yield.^[^ ^1^, ^2^, ^3^ ^]^ Under low‐nitrogen conditions, soybean plants interact with nitrogen‐fixing bacteria, such as rhizobia, to initiate nodulation in their roots. Nodulation is a dynamic and complex process in which physiological and cellular reprogramming occurs, leading to rhizobial infection and the formation of symbiotic organs and root nodules.^[^ ^4^, ^5^, ^6^, ^7^, ^8^, ^9^ ^]^ Significant progress has been made in elucidating the molecular mechanism underlying soybean nodulation. This includes the perception of rhizobial Nod factors (NFs) by the NF receptor (*GmNFR1α* and *GmNFR5α*) complex and the subsequent activation of nodulation signal transduction, which involves several essential core nodulation genes, such as *GmNSP1a/b* and *GmNINs*.^[^ ^10^, ^11^, ^12^, ^13^, ^14^, ^15^ ^]^ Previous studies have analyzed nodulation‐related gene expression profiles in root hairs and root cells at different time points during the nodulation process,^[^ ^16^, ^17^, ^18^ ^]^ highlighting that transcriptome changes constitute crucial molecular signatures for nodulation in roots. The majority of the genes whose expression is altered during nodulation are associated with signal transduction, basic metabolism, plant defense responses and cell modifications.^[^ ^19^, ^20^, ^21^, ^22^, ^23^ ^]^ Interestingly, many nodulation genes exhibit cell‐ or tissue‐specific regulation,^[^ ^8^, ^16^, ^17^, ^18^ ^]^ demonstrating that nodulation is a cell/tissue‐specific process in legumes.

Symbiotic nitrogen fixation has high energy requirements and consumes a large amount of photosynthates.^[^ ^24^, ^25^, ^26^, ^27^, ^28^ ^]^ Therefore, soybean plants undergo adaptive changes in their below‐ and aboveground organs to ensure optimal nodulation and plant growth. These plants have developed the autoregulation of the nodulation (AON) pathway, a long‐distance feedback loop involving the shoot. As nodule primordia initiate, *GmRIC1* and *GmRIC2* are activated by *GmNIN1a* and miR172c, leading to the production of the mobile CLE peptides GmRIC1 and GmRIC2. These peptides are subsequently transported from the roots to the shoots to activate the *Glycine max* nodule autoregulation receptor kinase GmNARK.^[^ ^29^, ^30^, ^31^, ^32^, ^33^, ^34^ ^]^ GmNARK, an LRR‐receptor kinase, systemically suppresses nodulation signaling activity in roots through shoot‐derived signals, such as microRNA2111 (miR2111), thereby preventing excessive nodule formation.^[^ ^29^, ^30^, ^35^ ^]^ This phenomenon suggests that profound molecular changes in shoots occur shortly after rhizobial infection to maintain the balance between nodulation and plant growth. Notably, phenotypic analysis revealed a substantial reduction in plant growth, along with the supernodulating phenotype exhibited by the GmNARK knockout mutant *nts1007*,^[^ ^30^ ^]^ indicating the crucial role of this gene in orchestrating the intricate balance of nodulation and plant growth balance. A previous study attempting to identify the shoot‐derived signals responsible for GmNARK‐mediated nodulation suppression revealed significant alterations in gene expression in the leaves of nodulated soybean plants,^[^ ^36^ ^]^ which has implications for our understanding of systemic modulation in response to root nodulation. However, the molecular changes that occur across all organs and the relationship among these organs have not been elucidated.

While the aforementioned studies have focused mainly on protein‐coding genes, the function of noncoding RNAs (ncRNAs) in various organs during nodulation remains poorly understood. These ncRNAs include miRNAs, long noncoding RNAs (lncRNAs) and circular RNAs (circRNAs), which participate in various biological processes by controlling the transcription and post transcription of downstream genes.^[^ ^37^, ^38^, ^39^ ^]^ Among the investigations of these ncRNAs, the functions of miRNAs have received significant attention during nodulation.^[^ ^40^, ^41^ ^]^ Several miRNAs, such as miR172, regulate nodulation by suppressing their target genes, which are associated with the auxin response and growth of plants.^[^ ^33^, ^34^, ^35^, ^42^, ^43^, ^44^ ^]^ Notably, our previous studies have identified miR172c as a crucial regulator of nodulation. miR172c is activated by *GmNINa* in roots to control every stage of nodulation from rhizobia infection to nodule morphogenesis by cleaving the mRNAs of the AP2 transcription repressor *GmNNC1*.^[^ ^33^, ^34^ ^]^ Additionally, the miR172c‐NNC1 module can activate AON by activating *GmRIC1/2*, which is suppressed by GmNARK and AON to maintain nodule homeostasis.^[^ ^33^, ^34^ ^]^ Strikingly, we recently showed that miR172c acts as a symbiosis‐specific signal, together with miR172 induced by fixed nitrogen, to accelerate the flowering of nodulated plants.^[^ ^45^ ^]^ These findings suggest that miR172c not only functions locally in root nodulation but also operates as a systemic signal to regulate plant growth. However, the precise regulatory mechanism governing the activity and dynamics of miR172c remains incompletely understood.

LncRNAs are crucial regulators of various biological processes.^[^ ^38^, ^46^ ^]^ Both lncRNAs and circRNAs can modulate the expression of downstream genes by acting as miRNA target mimics, thereby inhibiting the functions of miRNAs.^[^ ^47^, ^48^, ^49^, ^50^ ^]^ Many lncRNAs and circRNAs have been identified in soybean,^[^ ^51^, ^52^, ^53^, ^54^ ^]^ and several lncRNAs, such as *GmENOD40*, are differentially expressed during nodule development.^[^ ^55^, ^56^ ^]^ However, the exact roles of lncRNAs and circRNAs in nodulation remain uncharacterized, and importantly, the systemic regulation of ncRNAs during nodulation and plant growth is not known. Recently, competing endogenous RNA (ceRNA) networks have been shown that link the function of protein‐coding mRNAs with that of ncRNAs to regulate various biological processes.^[^ ^46^, ^47^, ^48^, ^49^, ^50^, ^56^ ^]^ Given that any transcripts harboring miRNA response elements can theoretically function as ceRNAs, these transcripts may represent a widespread form of posttranscriptional regulation of gene expression. However, no ceRNA regulatory network has been described for nodulation, despite the presence of key miRNA regulators such as miR172c in soybean.

Here, we performed comprehensive analyses of the regulation of protein‐coding and noncoding RNAs (ncRNAs) in roots, cotyledons and leaves at the stages of nodule formation and AON activation, leading to the identification of various classes of nodulation‐regulated RNAs (NR‐RNAs) and both nodulation‐ and GmNARK‐regulated RNAs (NNR‐RNAs) involved in the response to nodulation. We found that these NR‐ and NNR‐RNAs exhibited an organ‐specific pattern, and that roots and leaves presented the greatest number of NR‐ and NNR‐related RNAs and dynamic NR‐lncRNA‒miRNA‒mRNA crosstalk. Furthermore, we showed that lnc‐NNR6788 and lnc‐NNR7059 positively regulate nodulation through their target genes and that lnc‐NNR4481 negatively regulates miR172c and nodulation via a ceRNA axis. Thus, our study reveals comprehensive transcriptomic reprogramming at both the organ and organismal levels. This atlas can serve as a valuable resource and provides a framework for further functional and mechanistic analyses of these mRNAs and ncRNAs especially lncRNAs in the local and systemic regulation of nodulation, ensuring optimal nodulation and growth in soybean plants.

## 2 Results

### 2.1 Global Transcriptome Characterization of Multiple Tissues during Nodulation

**Figure 1:**
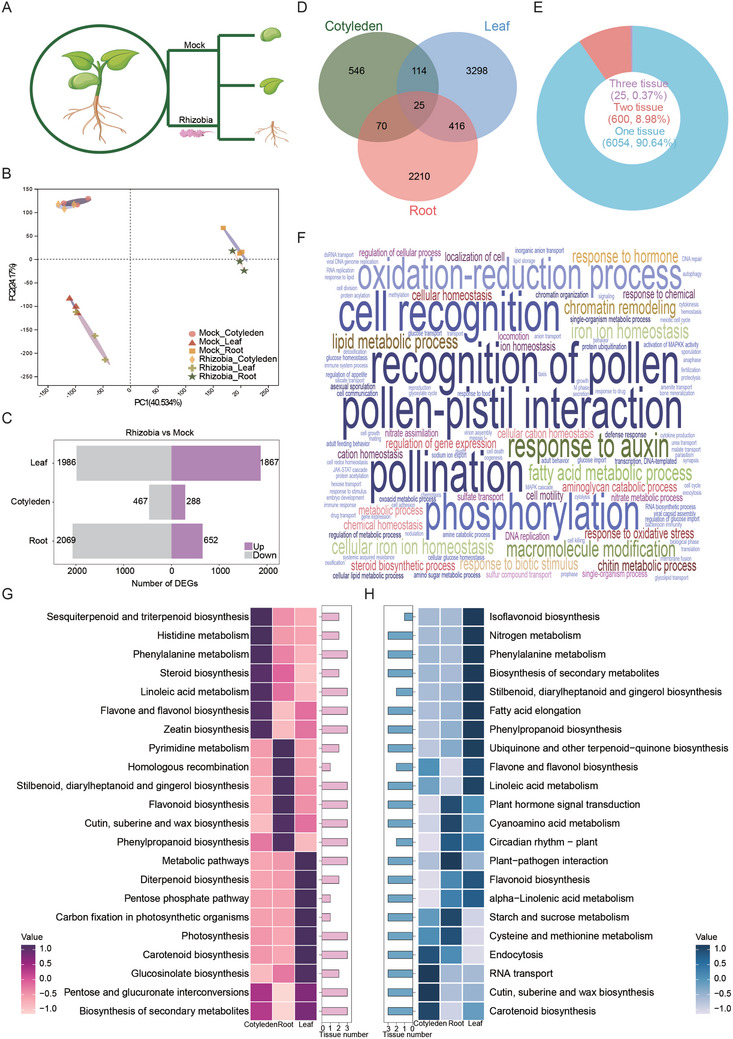
Global transcriptome characterization of multiple tissues during nodulation. A) Three tissues (cotyledon: C, leaf: L and root: R) were collected at 4 days after inoculated without (Mock) or with rhizobia. B) Principal component analysis (PCA) for 18 samples based on all gene expression. C) Number of differentially expressed mRNAs by comparing the rhizobia infected group to the mock group. The numbers of upregulated or downregulated mRNAs across all tested tissues. D) The distribution of nodulation‐regulated (NR)‐mRNAs in various tissues. E) Percent of the nodulation‐correlated mRNA that are identified in one, two and three tissues. F) Top biological processes (BPs) ranked by the number of NR‐mRNAs associated with each BP. The rank of BP is reflected by the size of the letters. G,H) Top 20 BPs that are commonly enriched in three tissues in the upregulated NR‐mRNAs (G) and in the downregulated NR‐mRNAs (H). Histogram indicates the number of tissues with functional enrichment for the corresponding BP.

To systemically profile the transcriptome changes during nodulation in an organism‐wide manner, we conducted RNA sequencing (RNA‐seq) on 3 different organs (roots, cotyledons and leaves), of 4‐day‐old soybean seedlings in the wild type Bragg cultivar and the loss‐of‐function GmNARK mutant *nts1007* at 4 days after rhizobia inoculation, when nodule primordia formation and AON activation occur (Figure **1**A). We obtained a total of 466.5 G data from 36 samples (18 samples from each genotype) against the soybean genome (*Glycine max*) to quantify the expression of both mRNAs and ncRNAs (Table S1A, Supporting Information). A gene with fragments per kilobase per million (FPKM) ≥ 0.5 in at least 10% of the samples was considered detectable in our dataset.

Using these criteria, we first analyzed the correlation among samples to determine the impact of rhizobia infection on the WT samples at this particular stage (Figure S1A and Table S1B, Supporting Information). We identified a total of 88,814 qualified genes across the examined tissues of the wild type plants (Table S1C, Supporting Information). Principal component analysis (PCA) revealed that these transcriptomes were primarily grouped on the basis of their organ identity (Figure 1B), suggesting that rhizobia infection leads to comprehensive reprogramming of gene expression, with organ identity being the primary factor influencing gene expression during nodulation at this stage. To examine how rhizobia infection may affect gene expression across different tissues, we identified the different expression genes (DEGs) by comparing the rhizobium‐infected group to the Mock group, with a cutoff of *q* value ≤ 0.05 and log_2_‐fold change ≥ 1, leading to the identification of 6,679 Nodulation‐Regulated mRNAs (NR‐mRNAs) in three organs (Figure S1B and Table S2A, Supporting Information). The greatest number of DEGs was detected in leaves (3,853), followed by roots (2,721) and cotyledons (755) (Figure 1C and Figure S1C–E, Supporting Information). The majority of these NR‐mRNAs (90.64%) were identified in a tissue‐specific manner, whereas only a small fraction (0.37%) was found in multiple tissues (Figure 1D,E and Table S2B, Supporting Information), further supporting the organ‐specific nature of nodulation.

The most pronounced pathways enriched by these NR‐mRNAs were associated with cell recognition, response to hormone (e.g., jasmonic acid, JA and auxin)‐related pathways, oxidation‒reduction processes, and phosphorylation, and this feature was recapitulated in multiple tissues (Figure 1F). Furthermore, the immune response, response to stress and cytoskeleton significantly changed in all three tissues (Figure S1F, Supporting Information). Rhizobia infection mainly affected secondary metabolites in cotyledons, plant hormone signal transduction in leaves, and plant‒pathogen interaction in roots (Figure S1G, Supporting Information). We identified the Top 20 enriched Kyoto Encyclopedia of Genes and Genomes (KEGG) pathways (Figure 1G,H). Notably, the pathways associated with the up‐ and downregulated genes during nodulation were more tissue‐specific and were frequently linked to the biological functions of tissues (Figure 1G,H). For example, the pathways associated with the biosynthesis of glucosinolates and diterpenoids were specifically enriched in genes associated with upregulated nodulation in leaves, suggesting an intensified response to rhizobia infection. In contrast, the processes of linolenic acid metabolism and phenylpropanoid biosynthesis were more abundant among the genes whose expression was upregulated in the cotyledons and roots, respectively, indicating enhanced plant growth at this stage. Interestingly, the majority of the Top 20 biological processes, including starch and sucrose metabolism, plant‒pathogen interaction, and flavonoid biosynthesis, were significantly enriched in the nodulation‐repressed genes in roots, but not those in cotyledons and leaves (Figure 1H), suggesting a decline in the functional capacity of infected roots in terms of energy metabolism, and rhizobia‒host communication.

### 2.2 Identification of NR‐ncRNAs Across Different Organs

**Figure 2:**
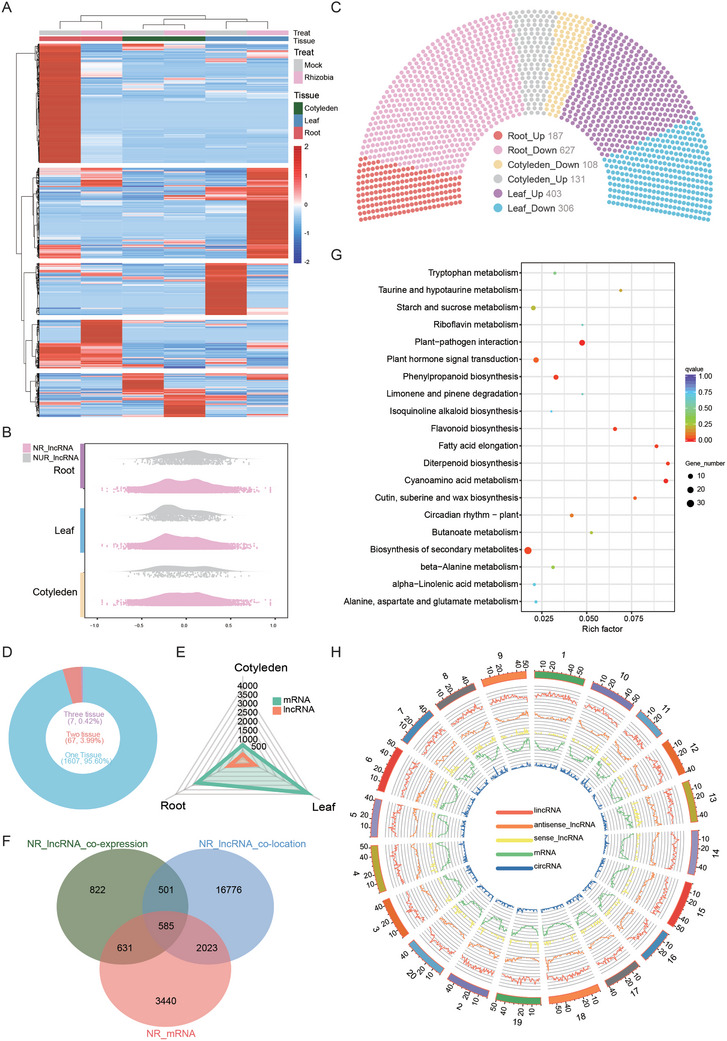
Identification and functional annotation nodulation‐regulated ncRNAs. A) The expression pattern of Nodulation‐Related lncRNAs (NR‐lncRNAs) during nodulation (*q* value ≤0.05; log_2_FC ≥ 1). B) The distribution of correlation coefficient between lncRNA expression and nodulation in each tissue. C) The distribution of NR‐lncRNAs in all examined organs. Each dot represents an NR‐lncRNA. D) Percent of the nodulation‐correlated lncRNAs that are identified in one, two and three tissues. E) Number of NR‐lncRNAs and mRNAs uniquely discovered in different tissues. F) Comparison of NR‐mRNAs and mRNAs associated with NR‐lncRNA. G) GO functional enrichment analysis of overlapping parts NR‐mRNAs and NR‐lncRNAs associated mRNAs. H) The distribution of non‐coding RNAs and mRNAs on chromosomes.

The tissue‐specific nature of nodulation inspired us to further examine the spatial landscapes of lncRNAs during this crucial phase of nodulation because lncRNAs exhibit more cell type‐specific expression patterns than mRNAs do and may play a significant role in the tissue‐specific nature of nodulation. We identified 55,172 lncRNAs and analyzed their tissue specificity (Table S3, Supporting Information). These lncRNAs were mainly composed of lincRNAs (50.4%) and antisense RNAs (23.6%) (Figure S2A, Supporting Information), and further analysis revealed that 2,387 lncRNAs may encode proteins (Figure S2B, Supporting Information). Moreover, we identified 2,427 novel lncRNAs and 402 mRNAs by comparing the transcript length, exon count, and open reading frame (ORF) length (Figure S2C–E, Supporting Information). We compared the lncRNA expression in the rhizobium‐infected group with that in the Mock group and identified 1,682 nodulation‐regulated lncRNAs (NR‐lncRNAs), with *q* value ≤0.05; log_2_FC ≥ 1 standard (Figure **2**A and Table S3A, Supporting Information). As expected, the NR‐lncRNAs, compared with nodulation‐unregulated lncRNAs (NUR‐lncRNAs), the NR‐lncRNAs were more strongly correlated with nodulation (Figure 2B and Table S3B, Supporting Information). Notably, the inoculated roots presented the greatest number of NR‐lncRNAs among the three examined tissues (Figure 2C). The roots and leaves expressed 814 and 709 NR‐lncRNAs, respectively, whereas the cotyledons expressed only 239 NR‐lncRNAs (Figure 2C). The observation of a significantly greater abundance of NR‐mRNAs (Figure S1B, Supporting Information) and NR‐lncRNAs (Figure 2A) in both roots and leaves suggested that these organs likely undergo more dramatic functional alterations than cotyledons do and thereby more significantly contribute to the establishment of symbiotic nodulation. We then investigated the tissue‐specific nature of nodulation by examining the number of NR‐lncRNAs shared by the three organs. Approximately 95% of the NR‐lncRNAs passed our nodulation‐regulation criteria exclusively in a single organ (Figure 2D,E), whereas only 4.4% of the NR‐lncRNAs were commonly regulated in more than one organ (Figure 2D).

To explore the potential functional role of these NR‐lncRNAs, we conducted a co‐expression analysis between the NR‐lncRNAs and all the mRNAs in each tissue, and check the intersection as the true target gene for NR‐lncRNAs (Figure 2F). For each NR‐lncRNA, we analyzed the biological process enrichment of the correlated mRNAs as an indicator of the NR‐lncRNA function. At the organismal level, the predominant enriched biological processes were related to the rhizobial response, such as pathogen interaction, flavone biosynthesis and wax biosynthesis (Figure 2G). These biological processes were also predominant among those that overlapped in the three tissues (Figure S2G, Supporting Information). At the tissue level, NR‐lncRNAs in roots are more closely associated with infection‐related biological processes than are those in cotyledons and leaves. For example, 73 NR‐lncRNAs were linked to plant defense‐related processes in the roots (Figure S2F and Table S3C, Supporting Information), and this was the highest quantity among the three examined tissues. The Top biological processes in the roots when ranked by the number of lncRNAs in each biological process, were related primarily to plant‒pathogen interaction (Figure S2F, Supporting Information). Overall, our lncRNA‒mRNA co‐expression analysis revealed that NR‐lncRNAs were functionally connected to the plant response to rhizobia infection at the organismal level, and such connections were particularly strong in roots.

To investigate whether and how alternative splicing (AS) is regulated during nodulation, we used RNA‐seq short reads to quantify AS events and conducted differential transcript splicing analysis among the three tissues. Five typical and distinct AS events, namely, alternative 3′ splice site (A3SS), intron retention (IR), kipped exon (SE), alternative 5′ splice site (A5SS) and mutually exclusive exon (MXE), were identified in each tissue when comparing the Mock and infection groups. After filtering with the criteria of a false discovery rate (FDR) ≤ 0.05 and percent spliced in difference (∆PSI) > 0.1, 2,075 differential splicing events from 242,931 loci were identified. The number of alternatively spliced events dramatically increased in the roots (Figure S2G, Supporting Information). Significant changes were detected in SE events on chromosome 11 (Figure S2H, Supporting Information) and in the A3SS on chromosome 13 (Figure S2I and Table S4, Supporting Information).

Interestingly, our analysis has revealed the presence of 758 circRNAs (Table S5A, Supporting Information), analyzed the sources of the circRNAs in all the samples, and found that the majority of the roots circRNAs were distributed in intergenic regions (Figure S2J, Supporting Information). We identified two NR‐circRNAs, with *q* value ≤ 0.05; log_2_FC ≥ 0 standard that exhibited high enrichment and tissue specificity. One of these circRNAs, novel_circ_0001231, was specifically upregulated in roots and leaves and potentially targeted *GLYMA_04G000100*, which encodes the ribonuclease P/MRP subunit POP1, whereas the other circRNA, novel_circ_0000591, was specifically downregulated in the cotyledons of the inoculated plant and may target *GLYMA_17G137800*, which encodes a vacuolar processing enzyme (VPE) (Figure S2K and Table S5A, Supporting Information). Next, we annotated the positions of all the NR‐nc RNAs and mRNAs on the chromosomes. The expression of these NR‐ncRNAs on the chromosomes was similar at both the antisense and the mRNA positions (Figure 2H and Table S5B–D, Supporting Information), indicating a possible regulatory relationship between NR‐ncRNAs and mRNAs.

### 2.3 Identification of NR‐miRNAs in Three Organs

**Figure 3:**
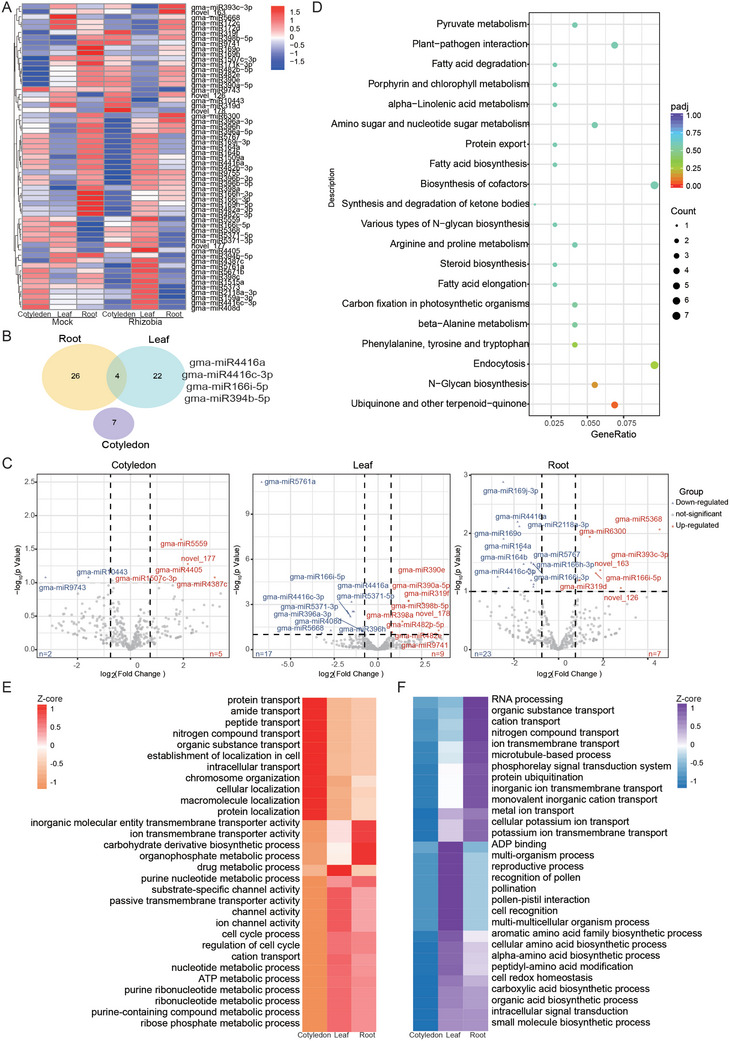
Identification of nodulation‐regulated (NR) miRNAs. A) The expression pattern of nodulation‐regulated miRNAs (NR‐miRNAs) during nodulation (*q* Value ≥ 0.1; log_2_FC ≥ 0.75). B) Veen shows the distribution of NR‐miRNAs in various tissues. C) The volcano plots show the downregulation of NR‐miRNAs in three tissues, marking the Top 10 differentially expressed miRNAs. (*q* Value ≥ 0.1; log_2_FC ≥ 0.75). D) KEGG functional enrichment of NR‐miRNAs target genes. E,F) Top 30 Biological processes (BPs) that are commonly enriched in three tissues in the upregulated NR‐miRNAs target genes (E) and commonly enriched in the downregulated NR‐miRNAs target genes (F). Histogram indicates the number of tissues with functional enrichment for the corresponding BP.

MiRNAs play a crucial role in nodulation locally and systemically through nodulation signaling and the AON pathway in soybean.^[^ ^33^, ^34^ ^]^ To investigate the expression profiles of miRNAs during nodulation, six small RNA libraries were retrieved via publicly accessible small RNA libraries, 505 known mature‐miRNAs and 493 pre‐miRNAs were mapped, and 99,989 species of sRNAs were discovered, totaling 26,982,247 (Table S6A, Supporting Information). We also identified novel miRNAs through database comparison, of which mature‐miRNAs accounted for 70, star‐miRNAs accounted for 37, and pre‐miRNAs accounted for 79 (Table S6A, Supporting Information). We generated a density map to visualize the miRNA distribution on chromosomes and found that the miRNAs were predominantly located on the positive strand (red outer circle) (Figure S3A, Supporting Information). Sequencing analysis revealed a total of 59 nodulation‐regulated miRNAs (NR‐miRNAs) in three tissues with *q* value ≤ 0.1; log_2_FC ≥ 0.75 standard (Figure **3**A; Table S6A, Supporting Information). Among these, the roots had a maximum of 26 NR‐miRNAs, whereas the leaves had 22. Four NR‐miRNAs were common to both roots and leaves, but the cotyledons had 7 NR‐miRNAs that were unique and not found in the roots or leaves (Figure 3B,C). Like NR‐miRNAs and NR‐mRNAs, NR‐miRNAs also exhibit distinct tissue specificity.

MiRNAs exert their effects by negatively regulating their target genes.^[^ ^37^ ^]^ Next, we conducted target prediction for these NR‐miRNAs, resulting in the identification of 756 candidate target mRNAs (Figure S3B and Table S6B, Supporting Information). KEGG analysis revealed that these target genes were involved mainly in ubiquinone and terpenoid–quinone biosynthesis, N‐glycan biosynthesis, endocytosis and plant‒pathogen interaction (Figure 3D). Furthermore, the analysis of the Top 30 Gene Ontology (GO) pathways revealed that in cotyledons, transport of protein, nitrogen compound, organic substance, chromosome organization, and cellular localization of protein and macromolecules regulated by miRNAs, the target genes were markedly upregulated, while in metal ion transport, biosynthetic processes of carboxylic acid and amino acids, cell redox homeostasis, and intracellular signal transduction, the target genes were significantly downregulated (Figure 3E,F). In leaves, there was an increase in drug metabolic process, cell cycle regulation, and ion transport and a decrease in multiorgan process, cell recognition, and pollen‒pistil interaction (Figure 3E,F). In roots, there was an increase in carbohydrate derivative biosynthetic process and transport of ion and inorganic molecules and a decrease in cation transport and nitrogen compound transport (Figure 3E,F). These results suggest that miRNAs play a crucial role in the tissue‐specific response to rhizobial infection in soybean.

### 2.4 GmNARK is Essential for NcRNA‐Mediated Nodulation

**Figure 4:**
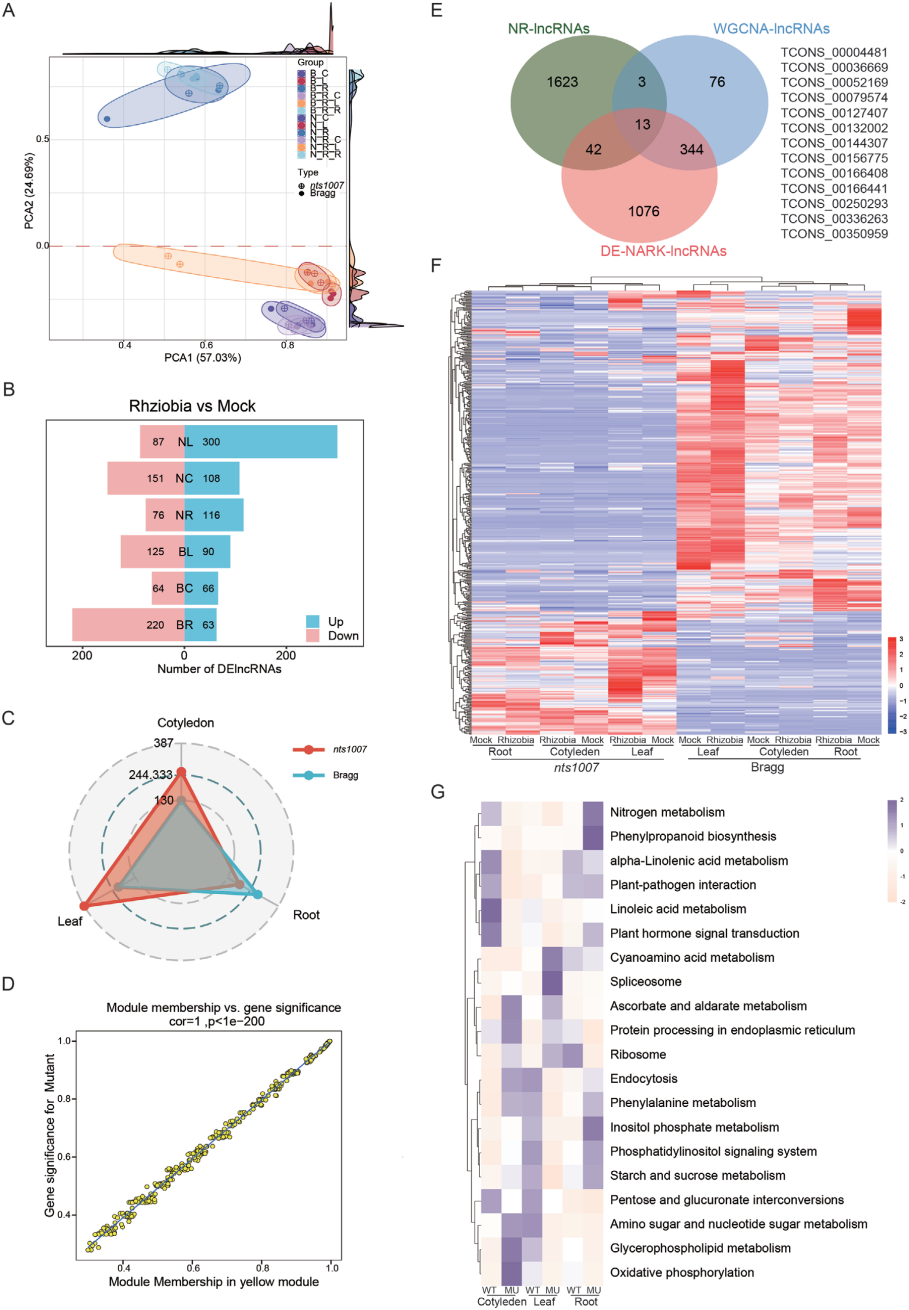
Identification of the GmNARK‐related lncRNAs. A) Principal component analysis (PCA) for 18 samples based on all gene expression. Side density map represents the degree of sample enrichment. B) Number of differentially expressed lncRNAs by comparing the rhizobia infected group to the mock group. The numbers of upregulated or downregulated lncRNAs across all tested tissues. B, Bragg; N, *nts1007*. C) Number of DE‐lncRNAs uniquely discovered in different tissues. D) The degree of association between genes and traits in MEyellow module. E) A Veen diagram shows the relationship between the different lncRNAs identified through diverse methods. F) The expression pattern of GmNARK related NR‐lncRNAs (NNR‐ lncRNAs). G) Top 20 KEGG pathways that are commonly enriched in different treated tissues NNR‐lncRNAs target genes. WT: Bragg; MU: *nts*1007.

GmNARK regulates nodulation in soybean locally and systemically.^[^ ^29^, ^30^ ^]^ The tissue‐specific patterns of NR‐ncRNAs led us to hypothesize that GmNARK plays a crucial role in NR‐ncRNA regulation. To test this possibility, we analyzed the mRNAs and ncRNAs of three tissues of the GmNARK mutant *nts1007* (N) and the wild‐type Bragg (B) at the same time point of nodulation (Figure 1A). PCA was used to identify the primary sources of variation among the samples (Figure **4**A). PC1/PC2 accounted for 81% of the total variation, indicating that the most significant differences were observed between the different tissues in both genotypes. The differences between the Bragg and *nts1007* mutants were more pronounced in the plants inoculated with rhizobia. Rhizobia infection had a stronger effect on *nts1007* plants in terms of nodulation responses. These results suggest a potential interaction between rhizobial infection and the AON pathway. In total, we identified 11,749 GmNARK‐ and nodulation‐regulated mRNAs (NNR‐mRNAs) at this particular stage of nodulation (Table S7A, Supporting Information). We observed minimal differences in the number of genes in cotyledons (3,324) but a significant change in the number of genes in the roots (3,639) and leaves (6,896) (Figure S4A,B, Supporting Information). These results indicate that GmNARK plays a more significant role in the aboveground portion of inoculated plants.

Next, we used two methods to identify lncRNAs associated with *GmNARK*. First, we identified 1477 lncRNAs (DE_NARK_lncRNAs) that were differentially expressed by comparing the significant changes in each tissue using DEGs (Mock and Rhizobia) (Figure 4B and Table S7B, Supporting Information). Similar to mRNAs, the aboveground parts (leaves and cotyledons) of the *nts1007* mutant presented a significant increase in the number of lncRNA variations (Figure 4C and Table S7C, Supporting Information). We then conducted weighted gene co‐expression network analysis (WGCNA) to investigate the correlation between different types (tissue, rhizobia infection and gene‐expression) and the expression of lncRNAs (Figure S4C, Supporting Information). Interestingly, we found a highly correlated module, MEyellow, with a correlation coefficient of 1.0 (Figure 4D). This module included 436 lncRNAs (WGCNA‐lncRNAs), and we observed that the expression patterns of all the genes were significantly downregulated in the *nts1007* mutant (Figure S4D and Table S7D, Supporting Information). When we compared the effects of *GmNARK* on the differential expression of lncRNAs, we found through a beanplot that the leaves and cotyledons of the mutant showed higher peaks and significant differences, while the roots mainly exhibited higher abundance of the expression (Figure S4E, Supporting Information). A comparative analysis of the NR‐lncRNAs, DE_NARK_lncRNAs, and WGCNA‐lncRNAs revealed the involvement of 13 lncRNAs in the hormone signaling and endocytosis pathways (Figure 4E,F), indicating that these lncRNAs are key genes that are jointly influenced by GmNARK and nodulation. We defined NNR‐lncRNAs as the union of DE_NARK_lncRNAs and WGCNA‐lncRNAs (Figure 4E,F and Table S7E, Supporting Information). These 1,554 NNR‐lncRNAs affect mainly sesquiterpenoid and triterpenoid biosynthesis, alpha‐linolenic acid metabolism and plant hormone signal transduction (Figure S4F and Table S7F, Supporting Information). In the cotyledons, phenylpropanoid biosynthesis, ribosome, nitrogen metabolism, amino sugar and nucleotide sugar metabolism and hormone signal transduction were strongly affected, while in the leaves, plant hormone signal transduction, endocytosis and amino sugar and nucleotide sugar metabolism were strongly affected. However, in the roots, the oxidative phosphorylation, linolenic acid metabolism, pentose and glucuronate interconversion and glycerophospholipid metabolism pathway were most significantly altered (Figure 4G and Table S7F, Supporting Information). These data suggest that GmNARK regulates nodulation through lncRNAs in different tissues to control diverse biological processes.

Moreover, we identified 33 NNR‐circRNAs using log_2_FC ≥ 1 and *q* value ≥ 0.1 standards (Figure S5A and Table S8, Supporting Information). These circRNAs are associated mainly with thiamine metabolism and terpenoid backbone biosynthesis, indicating the significant involvement of GmNARK in circRNA regulation. The same method was used to identify 114 DE‐NARK‐miRNAs and 37 WGCNA‐miRNAs in the MEgreen module (Figure S5B,C and Table S8A, Supporting Information), resulting in 4 miRNA intersections (Figure S5D, Supporting Information). Their union was then defined as 146 NNR‐miRNAs (Figure S5E and Table S8, Supporting Information). We identified the 5 miRNAs with the greatest differences among the three tissues in terms of quantity and abundance in cotyledons (Figure S5F, Supporting Information). This indirectly confirms the possibility that GmNARK plays a role in the aboveground portion. The target genes of NNR‐miRNAs were enriched mainly in N‐glycan biosynthesis and carbon fixation (Figure S5G, Supporting Information), suggesting that GmNARK primarily regulates nodulation via miRNAs to control protein glycosylation or lipid and carbon fixation.

### 2.5 Regulatory Network of NNR‐ncRNAs and NNR‐mRNAs

**Figure 5:**
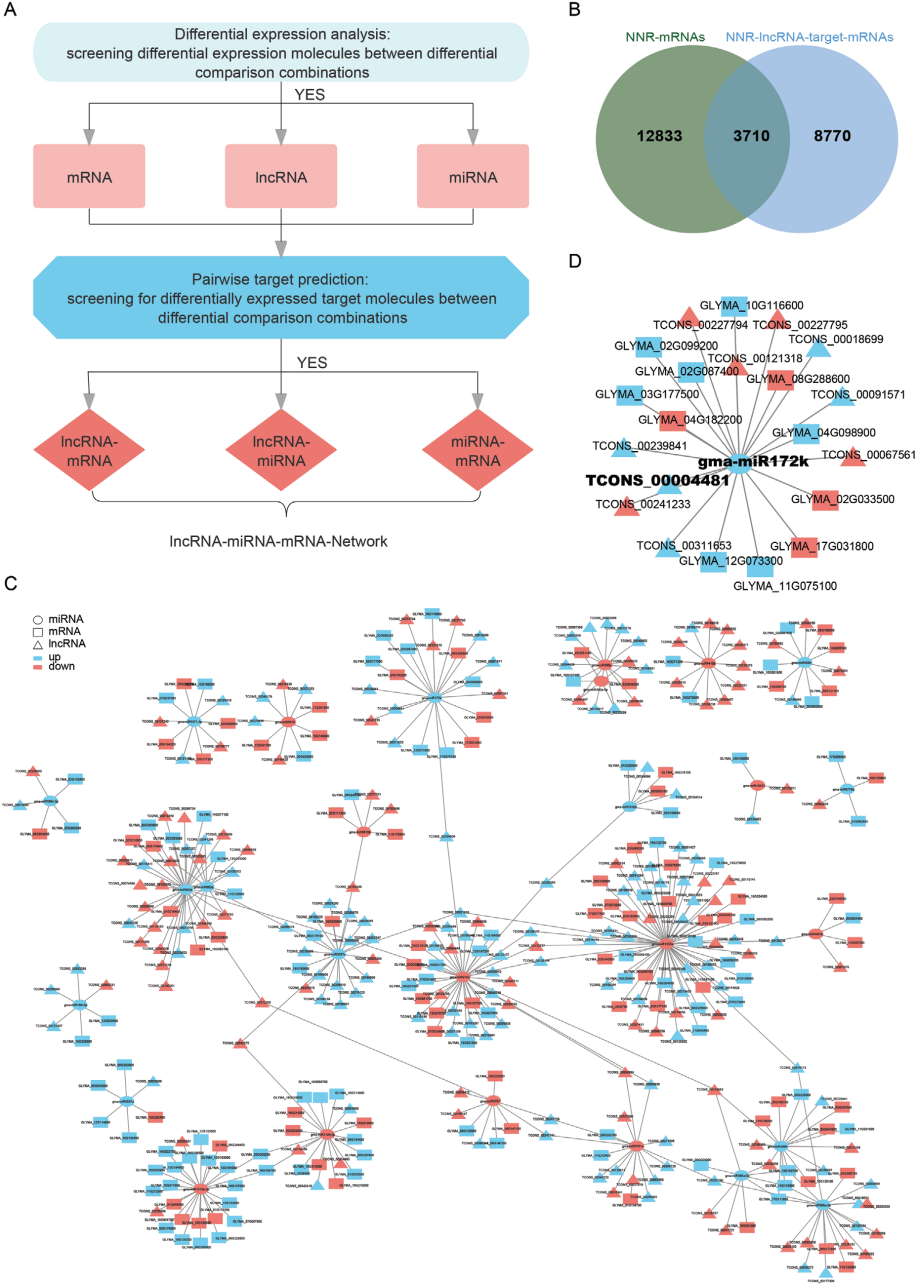
LncRNA‐miRNA‐mRNA network interaction analysis. A) Flowchart for the analysis of WTS (Whole Transcriptome Sequencing) data. B) A Veen diagram shows the relationship among the different NNR‐mRNA‐target‐mRNAs and NNR‐lncRNA‐target‐mRNAs. C) LncRNA‐miRNA‐mRNA network interaction diagram. Different shapes represent different types of RNA, while the colors red and blue indicate up‐ and down‐regulation, respectively. D) The network of miR172k and its target lncRNAs and mRNAs.

Recent studies have demonstrated that various regulatory RNAs with miRNA binding sites can competitively bind to the RNA‐induced silencing complex (RISC) composed of miRNAs to regulate mRNA levels.^[^ ^47^, ^48^, ^49^, ^50^ ^]^ To investigate the targeted regulatory relationships between various ncRNAs and mRNAs, as well as the interaction networks between these ncRNAs, we conducted a whole transcriptome sequencing (WTS) analysis. The targeted relationships of various differentially expressed RNAs between groups were analyzed based on previous targeted analysis and differential expression analysis between different combinations (Figure **5**A). Subsequently, regulatory networks involving the three RNA molecules were constructed. Because lncRNA regulate the mRNA expression of target genes through colocalization or coexpression,^[^ ^57^, ^58^ ^]^ we first performed an intersection analysis to compare the target genes of NNR‐lncRNAs and NNR‐mRNAs. When the co‐localized and co‐expressed NNR‐lncRNA target genes overlap with NNR‐mRNAs, there is a greater possibility that these differentially expressed mRNAs are directly or indirectly regulated by lncRNAs. Thus, we identified 3710 target mRNAs of NNR‐lncRNAs (Figure 5B, Table S9A, Supporting Information).

To investigate the function of NNR‐lncRNAs, we performed an integrative analysis to explore the interactions between lncRNAs and their target miRNAs, aiming to elucidate their functional associations. Initially, we identified 49,859 pairs by filtering out lncRNAs that may act as miRNA precursors the basis of homology. Simultaneously, we screened lncRNAs with miRNA binding sites as target lncRNAs for differentially expressed miRNAs, resulting in 1,359 pairs (Table S9B, Supporting Information). According to the competing endogenous RNA (ceRNA) theory, miRNAs and lncRNAs are competitively related, leading to a negative correlation in their expression levels.^[^ ^50^ ^]^ By comparing the differentially expressed miRNAs and differentially targeted lncRNAs across various combinations, we identified increased levels of miRNAs and decreased levels of targeted lncRNAs, as well as decreased levels of miRNAs and increased levels of targeted lncRNAs (Table S9C, Supporting Information). Additionally, we searched for lncRNA target gene pairs sharing the same miRNA binding site and subsequently established regulatory relationships among lncRNAs, miRNAs, and mRNAs, where lncRNAs act as decoys, miRNAs act as cores, and mRNAs act as targets, to construct a ceRNA regulatory network. At the transcriptional level, the ceRNA regulatory network analysis revealed the mechanism by which ncRNAs regulate gene expression (Figure 5C and Figure S6A; Table S10, Supporting Information).

MiR172 plays a crucial role in soybean nodulation. Interestingly, we observed a significant correlation between NNR‐lncRNA (TCONS_00004481, named lnc‐NNR4481), miR172k, another member of the miR172 family, and its predicted target mRNAs in the aboveground portion (Figure 5D). These target genes belong to different gene families and are associated with starch, sucrose, and α‐linolenic acid metabolism (Figure 5D, Figure S6B,C, Supporting Information), which significantly differed according to previous analyses. These findings suggest that lnc‐NNR4481‐miR172k may function through the regulation of energy and signaling related to sugars and hormones, such as JA. Similarly, we identified another significantly correlated gene pair, TCONS_00169915 (lnc‐NNR9915) and miR2111b (Figure S6D, Supporting Information), which mediate nodulation suppression through posttranscriptional regulation of the symbiosis suppressor *Too Much Love* (*GmTML*), a root‐active kelch‐repeat F‐box protein in infected roots, in *Lotus japonicus*.^[^ ^59^ ^]^ Interestingly, the predicted target gene of lnc‐NNR9915‐related miR2111b encodes a protein related to pectic arabinogalactan synthesis, suggesting that cell wall remodeling is highly likely to be regulated by the action of ceRNA during nodulation. Overall, our findings reveal the significant role of NNR‒lncRNA‒miRNA‒mRNA networks in the interaction between rhizobia and their host plants, as well as during nodulation.

### 2.6 Quantitative Analysis of the NR and NNR Gene Expression

**Figure 6:**
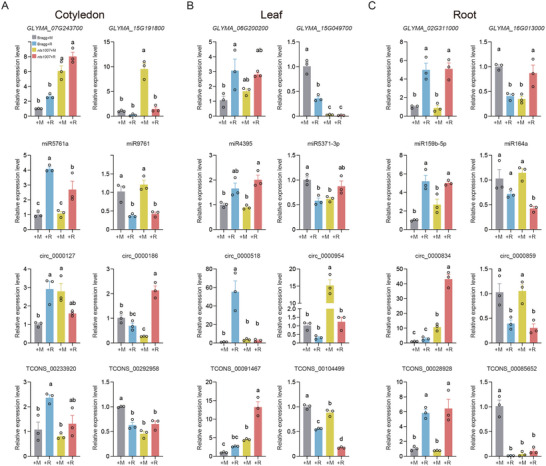
The expression verification of nodulation‐related mRNAs, miRNAs, circRNAs and lncRNAs in soybean. A–C) The expression levels of some novel nodulation‐regulated (NR) mRNAs, miRNAs, circRNAs and lncRNAs in cotyledon (A), leaf (B) and root (C) under the same conditions as RNA‐seq. Two genes in each type of RNAs were selected to validate the RNA‐seq results in each tissue (*n* = 3). Bragg+M: Bragg without inoculation, Bragg+R: Bragg with inoculation, *nts1007*+M: *nts1007* without inoculation, *nts1007*+R: *nts1007* with inoculation. The experiments were repeated for three times. All data are presented as means ± SEM from three biological repeats. Different letters indicate significant differences at *p* < 0.05 (One‐way ANOVA).

Our investigation of nodulation revealed an intricate regulatory network that encompasses a wide array of coding and noncoding genes. Moreover, our transcriptome analysis revealed numerous novel mRNAs, miRNAs, circRNAs, and lncRNAs potentially involved in symbiotic nitrogen fixation. To further characterize the expression patterns of the candidate genes in various tissues during nodulation and validate our profiling results, we inoculated 4‐day‐old Bragg and *nts1007* plants with the rhizobium USDA110. The leaves, cotyledons, and roots samples subsequently collected at 4 days after inoculation (DAI) under the same conditions as those used for RNA‐seq were subjected to qRT‒PCR analysis. Two mRNAs were selected from each tissue as candidates, one upregulated and another downregulated after inoculation, based on their FPKM values in the transcriptome (Figure S7 and Table S11, Supporting Information). The same criteria were applied to the miRNA, circRNA and lncRNA data, resulting in the selection of 24 genes as candidates for qRT‒PCR analysis (Figure S7A–C, Supporting Information). The relative expression levels of these candidates generally matched their FPKM or Transcripts Per Million (TPM) values in the transcriptome (Figures **6** and S7, Supporting Information), validating the reliability of our RNA‐seq data.

Among these genes, the majority were up‐ or downregulated by rhizobial infection in a GmNARK‐dependent manner. Some of these genes exhibited organ‐specific expression patterns in response to rhizobial infection (Figure 6 and Figure S7D, Supporting Information). For instance, the expression of *GLYMA_02G311000*, *GLYMA_06G200200*, miR159b‐5p and TCONS_00028928 was significantly upregulated after rhizobial inoculation in the roots in a GmNARK‐independent manner, as demonstrated by the qPCR and sequencing results (Figure 6A–C). In contrast, circ_0000518 was highly induced by rhizobia in the leaves in a GmNARK‐dependent manner. Furthermore, the expression levels of *GLYMA_07g243700*, circ_000843, circ_000186 and TCONS_00091467 in various organs of the *nts1007* mutant were significantly greater than those in the wild type after rhizobial inoculation (Figure 6), suggesting that these genes were negatively regulated by *GmNARK* at the organismal level. Overall, these data demonstrate that the expression of a diverse set of genes in various tissues is affected at the early stage of nodulation and that major transcriptional changes in various tissues are regulated by GmNARK. Given that lncRNAs exhibit the most notable alterations at this stage of nodulation and are related to both mRNAs and miRNAs, we subsequently concentrated on exploring the functions and characteristics of NNR‐lncRNA genes in subsequent experiments.

### 2.7 Lnc‐NNR6788 and Lnc‐NNR7059 Positively Regulate Nodulation by Targeting MRNAs

**Figure 7:**
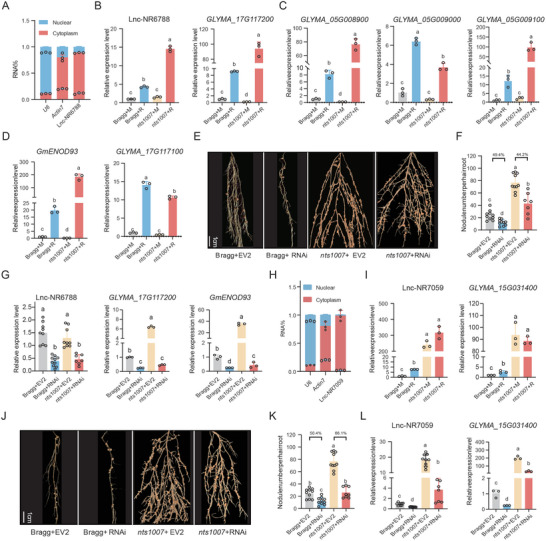
Lnc‐NNR6788 and lnc‐NNR7059 increase the number of nodules by regulating their target genes. A) The qRT‐PCR analysis of the lnc‐NR6788 RNA in both nucleus and cytoplasm. Nuclear *U6* and cytoplasmic *Actin7* mRNA served as controls (n = 3). B,C,D) The expression levels of lnc‐NR6788, its target gene *GLYMA_17G117200* (B) and homologous genes of *GLYMA_17G11720* at soybean. (C,D) in the roots under the same conditions as RNA‐seq (*n* = 3). Bragg+M: Bragg without inoculation, Bragg+R: Bragg with inoculation, *nts1007*+M: *nts1007* without inoculation, *nts1007*+R: *nts1007* with inoculation. E,F) Nodulation phenotype (E) and nodule number per hairy roots (F) (*n* = 10) of Bragg/*nts1007* plants expressing EV2 (pGFP‐930) and lnc‐NR6788‐RNAi at 21 DAI. Scale bar = 1 cm. G) The expression levels of lnc‐NR6788, *GmENOD93*, and *GLYMA_17G117200* in individual hairy roots expressing EV2 and RNAi at 21 DAI (*n* = 3). H) The qRT‐PCR analysis of the lnc‐NR7059 RNA in both nucleus and cytoplasm. Nuclear *U6* and cytoplasmic *Actin7* mRNA served as controls (*n* = 3). I) The expression levels of lnc‐NR7059 and *GLYMA_15G031400* in root. Bragg+M: Bragg without inoculation, Bragg+R: Bragg with inoculation, *nts1007*+M: *nts1007* without inoculation, *nts1007*+R: *nts1007* with inoculation. J,K) Nodulation phenotype (J) and nodule number per hairy roots (K) (*n* = 8–10) of Bragg plants expressing EV2 (pGFP‐930) and lnc‐NR7059‐RNAi (RNAi) at 21 DAI. Scale bar = 1 cm. L) The expression levels of lnc‐NR7059 and *GLYMA_15G031400* in individual hairy roots expressing EV2 and RNAi at 21 DAI (*n* = 3–9). The experiments were repeated for three times. All data are presented as means ± SEM. Different letters indicate significant differences at *p* < 0.05 (One‐way ANOVA).

To experimentally validate that these lncRNAs are involved in nodulation, we first selected NR‐lncRNA, TCONS_00116788 (initially named as lnc‐NR6788, Table S7B, Supporting Information) which targets a homolog gene (*GLMA_17G117200*) of *GmENOD93* that promotes nodulation for functional characterization,^[^ ^60^ ^]^ for further characterization. In addition to *GLMA_17G117200*, which showed the highest abundance and strongest correlation with lnc‐NR6788, four genes (*GLYMA_05G008900*, *GLYMA_05G009000*, *GLYMA_05G009100* and *GmENOD93*) with the highest homology to *GLMA_17G117200* were also selected for the subsequent experiments (Figure S8A,B and Table S12A, Supporting Information). To elucidate the molecular mechanisms that govern the role of lnc‐NR6788 in nodulation, we first sought to examine the subcellular localization of lnc‐NR6788 in soybean roots. This step is crucial, as the subcellular localization of lncRNAs dictates their regulatory modes.^[^ ^61^ ^]^ The qRT‒PCR analysis of the relative expression of lnc‐NR6788 in the nucleus and cytoplasm revealed that lnc‐NR6788 was predominantly localized in the cytoplasm (Figure **7**A), which is consistent with the bioinformatic prediction via lncATLAS (https://lncatlas.crg.eu/). These findings indicate that lnc‐NR6788 may play a pivotal role in regulating the stability, translation, and signaling of its target mRNAs. The expression of lnc‐NR6788, its target gene (*GLMA_17G117200*) and other target gene homologues was induced by rhizobia in roots, and their induction was further enhanced in the GmNARK mutant roots (Figure 7B–D). These results suggest that lnc‐NR6788 may exert its regulatory function by targeting *GLMA_17G117200* and its homologous genes under the regulation of GmNARK. Next, we used RNA interference (RNAi) methodology to suppress the expression of the lncRNA and subsequently assessed the nodule number in lnc‐NR6788‐RNAi hairy roots at 28 DAI. Knockdown of lnc‐NR6788 resulted in a significant reduction (49.4%) in nodule number in the WT, and a substantial decrease of approximately 44.2% in the nodule number per hairy root compared with the vector‐control (EV2) roots in *nts1007* (Figure 7E,F and Table S12B, Supporting Information). Accordingly, the expression of lnc‐NR6788 and two target genes was also downregulated in both the WT and *nts1007* mutant (Figure 7G). These results indicate that lnc‐NR6788 is a positive regulator of nodulation, acting downstream of *GmNARK*.

Using a similar approach, we analyzed another type of NR‐lncRNA, TCONS_00067059 (originally named as lnc‐NR7059, Table S7B, Supporting Information), which targets the unknown function mRNA, *GLYMA_15G031400* (Figure S8C, Supporting Information), possibly similar to its homologous gene *GmCHG* (Figure S8D, Supporting Information), by encoding a beta‐glucosidase to regulate the release of free isoflavones from their conjugate forms.^[^ ^62^, ^63^ ^]^ We found that lnc‐NR7059 is also localized in the cytoplasm (Figure 7H). The expression levels of lnc‐NR7059 and *GLYMA_15G031400* were significantly influenced by rhizobia and GmNARK (Figure 7I). The functional analysis results showed that the knockdown of lnc‐NR7059 decreased the number of nodules in *nts1007* mutant by 66% (Figure 7J–L and Table S12B, Supporting Information). These results suggest that lnc‐NR7059 plays a crucial role in promoting nodulation in a GmNARK‐dependent manner. Taken together, our data identified two important NNR‐lncRNAs that positively regulate nodulation homeostasis by targeting specific target mRNAs involved in nodulation, hereafter referred to as lnc‐NNR6788 and lnc‐NNR7059.

### 2.8 Lnc‐NNR4481 Negatively Regulates Nodulation by Modulating the MiR172c as a CeRNA

**Figure 8:**
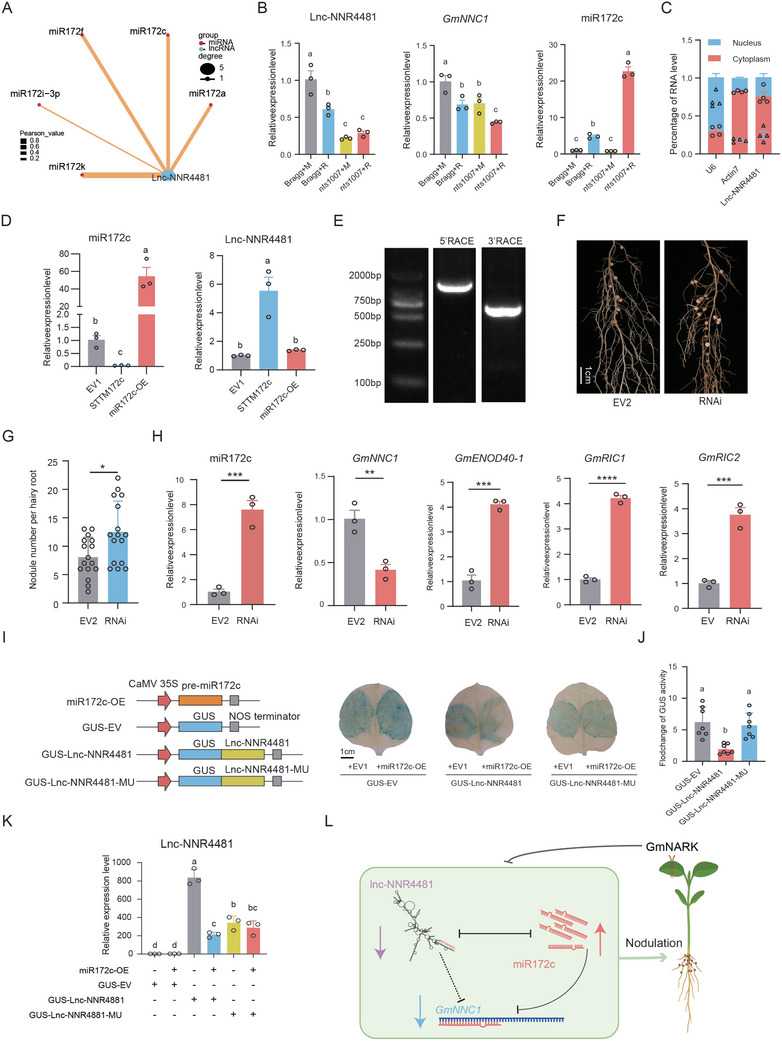
Lnc‐NNR4481 functions as a miRNA sponge for miR172c to regulate the expression of *GmNNC1* and subsequent nodulation. A) The correlation network involving lnc‐NNR4481 and 5 members of miR172 family. B) The expression levels of lnc‐NNR4481, *GmNNC1*and miR172c in the roots under the same conditions as RNA‐seq (*n* = 3). Bragg+R and Bragg+M: the wild type Bragg inoculated with and without rhizobia; *nts1007*+R, *nts1007*+M: *nts1007* mutant inoculated with and without rhizobia. C) The qRT‐PCR analysis of the lnc‐NNR4481 RNA in both nucleus and cytoplasm. Nuclear *U6* and cytoplasmic *Actin7* mRNA served as controls (*n* = 4). D) The expression levels lnc‐NNR4481 in individual hairy roots of Bragg overexpressing EV1 (pEGAD), *35S::STTM172c*, and *35S::pre‐miR172c* at 4 DAI (*n* = 3). E) The 5′ and 3′ end sequences of lnc‐NNR4481 were obtained. 5′ Rapid amplification of cDNA ends (5′ RACE) and 3′ RACE was designed from soybean roots. F,G) Nodulation phenotype (F) and nodule number per hairy roots (G) (*n* = 16) of Bragg plants expressing EV2 (pGFP‐930) and Lnc‐NNR4481‐RNAi (RNAi) at 21 DAI. Scale bar = 1 cm. H) The expression levels of miR172c, *GmNNC1*, *GmENOD40‐1*, *GmRIC1*, and *GmRIC2* in individual hairy roots expressing EV2 and RNAi at 21 DAI (*n* = 3). I,J) Histochemical GUS staining assay (I) and fluorometric GUS activity assay (J) illustrating the inhibitory effect of miR172c on lnc‐NNR4481 in *N. benthamiana* leaves (Scale bars = 1 cm). The construct containing empty vector (GUS‐EV), GUS‐Lnc‐NNR4481, or GUS‐Lnc‐NNR4481‐MU was co‐transformed with EV1 or *35S::pre‐miR172c* into epidermal cells of *N. benthamiana* leaves. The transgenic leaves were collected 48 h after transformation. Each column in (J) represents the GUS activity fold change of *N. benthamiana* leaves co‐transformed with *35S::pre‐miR172c* compared to EV1. Error bars representing SEM (*n* = 7). K) The expression level of lnc‐NNR4481 in *N. benthamiana* leaves (*n* = 3). The above experiments were repeated for three times. All data are presented as means ± SEM. Different letters indicate significant differences at *p* < 0.05 (One‐way ANOVA) (B,I,J,L) or at *p* < 0.05 (Mann–Whitney *U* test: ns, not significant; *p < 0.05; **p < 0.01; ***p < 0.001; ****p < 0.0001) (F,G,H,L) A proposed model illustrates the role of lnc‐NNR4481 in soybean nodulation. At the early stage of nodulation, rhizobia induce miR172c to alleviate its suppression on nodulation suppressor *GmNNC1* to promote nodulation. Meanwhile, lnc‐NNR 4481 is downregulated to promote nodulation by releasing miR172c, thereby accelerating nodulation. In infected roots, the lnc‐NNR4481 serves as a decoy for miR172c, effectively functioning as a sponge that sequesters this miRNA. This sequestration relieves the inhibitory effect of miR172c on *GmNNC1*, thereby fostering the downregulation of *GmNNC1* and maintaining a homeostatic balance in the number of root nodules. GmNARK in shoots modulates the lLnc‐NNR448‐miR172c‐*GmNNC1* module to control optimal nodule number (Created in BioRender.com).

Our transcriptomic analyses indicate that lnc‐NNR4481 may regulate nodulation through the miR172k‐mediated ceRNA regulatory axis. To prove this possibility, we first mapped the binding sites of miR172 family members to lnc‐NNR4481 and identified 5 candidates, including miR172c (**Figure** **8**A and Table S13A, Supporting Information), which is a key regulator of soybean nodulation.^[^ ^33^ ^]^ This finding suggests the potential regulatory role of lnc‐NNR4481 through miR172c and its target gene *GmNNC1*. The results show that, lnc‐NNR4481 and *GmNNC1* were significantly downregulated but miR172c upregulated, respectively, at 4 DAI in the infected roots, and their expression levels were oppositely regulated by *GmNARK* (Figure 8B), validating the expression patterns of these genes and their relationships with *GmNARK*. Further qRT‒PCR results showed that lnc‐NNR4481 is localized primarily in the cytoplasm (Figure 8C), supporting a potential association with miR172c. To confirm the regulatory interplay between lnc‐NNR4481 and miR172c, we analyzed the effect of miR172c on lnc‐NNR4481. Although the expression level of lnc‐NNR4481 did not significantly change in the transgenic roots overexpressing miR172c, it dramatically increased in the transgenic hairy roots with reduced activity of miR172c (Figure 8D). Moreover, a miR172c target site was found in the lnc‐NNR4481 region (Figure S9A, Supporting Information), suggesting that miR172c might have a negative effect on lnc‐NNR4481.

To explore the function of lnc‐NNR4481 in soybean nodulation, we obtained the 3′ and 5′ cDNA termini of lnc‐NNR4481 through the RNA ligase‐mediated rapid amplification of cDNA ends (RLM‐RACE) technique (Figure 8E), and subsequently determined or verified its full‐length sequence. We used RNAi technology to knockdown this gene and evaluated the nodule number of the lnc‐NNR4481‐RNAi hairy roots at 28 DAI (Figure 8F). Remarkably, the nodule number per lnc‐NNR4481‐RNAi hairy root was significantly greater than that of the vector control roots by approximately 55% (Figure 8F,G; Table S13B, Supporting Information), suggesting that lnc‐NNR4481 negatively regulates nodulation. The levels of the nodulation genes miR172c, *GmNNC1*, *GmENOD40‐1*, *GmRIC1* and *GmRIC2* in the roots of the lnc‐NNR4481‐RNAi transgenic plants were significantly increased, suggesting that lnc‐NNR4481 regulates nodulation by modulating the expression of miR172c and its downstream genes (Figure 8H).

Next, we conducted GUS assays to verify the relationship between lnc‐NNR4481 and miR172c in *Nicotiana benthamiana* leaves. GUS staining and GUS enzyme activity assays revealed that the coexpression of *35S::miR172c* and *35S::GUS‐lnc‐NNR4481* resulted in a significant reduction in GUS activity compared with that of the control, while this phenomenon completely disappeared in the *35S::GUS‐lnc‐NNR4481‐MU* context with six point mutations in the sequence matching for miR172c (Figure 8I,J and Figure S9A, Supporting Information). These findings suggest that the direct and specific binding of miR172c to lnc‐NNR4481 leads to its degradation. The expression of lnc‐NNR4481 in tobacco leaves significantly decreased following co‐transformation with miR172c (Figure 8K). In contrast, no significant change was observed in lnc‐NNR4481‐MU, indicating that miR172c mediated the downregulation of lnc‐NNR4481 expression through direct binding. These results suggest that lnc‐NNR4481 regulates nodulation through miR172c, which represses *GmNNC1* expression. Altogether, our findings suggest that lnc‐NNR4481 acts as a sponge for miR172c, thereby regulating *GmNNC1* expression and contributing to the promotion nodulation in soybean.

Previously, we have shown that miR172c is induced by rhizobia and acts as a long‐distance signal to promote nodulation‐accelerated flowering of soybean.^[^ ^64^ ^]^ To test whether lnc‐NNR4481 functions systemically and whether it is regulated by GmNARK, we collected roots, shoots and their phloem exudates(Figure S9B, Supporting Information) and analyzed the expression of lnc‐NNR4481 and miR172c as a control (Figure S9C,D, Supporting Information). After rhizobial inoculation, the abundance of miR172c increased in inoculated roots and root exudes (root phloem sap) of WT, and was further enhanced in the *nts1007* mutant (Figure S9C, Supporting Information), which is consistent with our previous results.^[^ ^64^ ^]^ Interestingly, we found that the expression levels of miR172c decreased in leaves, meanwhile the abundance of miR172c in *nts1007* significantly increased from the shoots to the roots (shoot phloem sap) compared with that in WT plants (Figure S9C, Supporting Information). These results suggest that GmNARK inhibits both upward and downward transport of miR172c, thereby maintaining the homeostasis of miR172c in leaves and roots, respectively. In contrast, the expression level of lnc‐NNR4481 in *nts1007* roots decreased, and the reduction in the abundance of lnc‐NNR4481 of exuding from the roots to shoots (root phloem sap) induced by rhizobia was completely abolished in *nts1007* mutant (Figure S9D, Supporting Information). These results indicate that lnc‐NNR4481 may act as a bidirectional signal during nodulation and that the upward and downward transport of lnc‐NNR4481 is controlled by GmNARK. Thus, GmNARK may systemically control nodulation homeostasis by modulating the long‐distance transport of these ncRNAs and maintaining the optimal levels of lnc‐NNR4481 and miR172c in roots.

## 3 Discussion

Symbiotic nodulation begins when insufficient nitrogen is available for the growth of germinating plants in the soil. Previous studies have focused predominantly on roots transcriptomic profiles at different stages of nodulation or on different cell types of roots,^[^ ^17^, ^65^ ^]^ revealing the dynamic molecular changes in soybean roots during nodulation. In addition to controlling local nodulation, plants require coordination of the growth and development of every organ. However, little is known about the transcriptional regulation of nodulation at the organismal level. Here, we used comprehensive RNA‐seq to study molecular changes in roots, cotyledons and leaves at the stage of nodule formation and AON activation in soybean. At this stage, we observed transcriptional reprogramming that encompassed all the organs of the nodulated soybean plants. We demonstrated that the transcriptional changes exhibited distinct organ‐specific patterns, but these three organs share very small portions of the transcriptome changes. Notably, despite their distinctness, the three organs exhibited minimal overlap in their transcriptome changes, suggesting a conserved organ‐specific role for the involved genes. Interestingly, the leaves exhibited the most profound transcriptome alterations among the three organs, followed by the roots, whereas the cotyledons displayed the least extent of alteration. This observation is conceivable because both the leaves and roots are rapidly developing organs at this stage, whereas cotyledons serve as organs that provide carbon and nutrients for the growth of other organs. Our analysis showed that, each organ exhibited a distinct change in biological processes, with leaves involved mainly in hormonal signaling transduction, roots in plant‐pathogen interaction, and cotyledons in secondary metabolism. Thus, these tissue‐specific transcriptional changes might contribute significantly to the coordinated regulation of nodulation and plant growth during this crucial developmental phase.

Transcriptional reprogramming is a prominent phenomenon observed during soybean nodulation. Although there has been significant attention given to alterations in the expression of protein‐coding genes,^[^ ^16^, ^17^, ^18^ ^]^ little is known about the transcriptional reprogramming of ncRNAs and the relationship between coding RNAs and ncRNAs. In particular, the transcriptomic alterations in all three organs of inoculated soybean plants during nodule formation and AON activation, aimed at understanding systemic regulatory mechanisms at both the organ and organismal levels, have not been investigated. Our data revealed that, in addition to coding RNAs, many ncRNAs, including lncRNAs, miRNAs and circRNAs, are significantly altered in all three organs at this particular stage. Importantly, the majority of NR‐coding RNAs and ncRNAs exhibit organ‐specific expression patterns, and some of them are co‐expressed, suggesting a complex regulatory network that involves both mRNAs and ncRNAs to precisely regulate the coordination of plant growth and nodulation in inoculated plants. However, these NR‐ncRNAs exhibit substantial quantitative disparities, with the highest abundance found in infected roots and the lowest in cotyledons. Among them, lncRNAs presented the most significant alteration during early nodulation. Notably, the expression of numerous lncRNAs is tightly regulated in an organ‐specific manner and is closely related to tissue‐specific biological processes. These observations indicate the extensive involvement of NR‐lncRNAs in transcriptional and posttranscriptional regulation, mediating both organ‐specific and organism‐wide modulation of nodulation and plant growth.

Furthermore, we identified many known (e.g., miR172, miR393 and miR2111) and novel miRNAs. Notably, we found that four NR‐miRNAs were shared by the roots and leaves, supporting the common roles of these NR‐miRNAs in modulating the growth of the roots and leaves. In contrast, all seven NR‐miRNAs are unique to cotyledons, suggesting their specific roles in processes unique to cotyledons. Unexpectedly, we identified only two NR‐circRNAs, one shared by the roots and leaves and the other in the cotyledons. This limited number of circRNAs may be attributed to the current limitations of circRNA identification techniques. Alternatively, circRNAs have a minor role in regulating organ‐ and organismal remodeling in inoculated soybean plants.

Plants regulate nodulation homeostasis via the AON pathway, which involves the activation of GmNARK in the shoots.^[^ ^36^ ^]^ A previous study showed that GmNARK plays a crucial role in transcriptionally regulating coding genes in the leaves of inoculated soybean plants.^[^ ^36^, ^66^ ^]^ In this study, we found that GmNARK mutant plants exhibited a greater abundance of differentially expressed mRNAs (DE‐mRNAs) and non‐coding RNAs (DE‐ncRNAs) during nodulation. This suggests that GmNARK controls the transcriptional homeostasis during soybean nodulation. Remarkably, GmNARK regulates the expression of numerous NR‐ncRNAs, with a greater number of NNR‐ncRNAs in cotyledons and leaves compared to roots during nodulation. Among these, NNR‐lncRNAs exhibited the highest abundance across various organs, particularly in cotyledons. The influence of GmNARK on phenylpropanoid biosynthesis was evident in cotyledons, while endocytosis was strongly affected in leaves. However, in roots, the oxidative phosphorylation pathway was most significantly altered. These observations suggest that GmNARK may play a pivotal role in systematically regulating early nodulation across multiple tissues through lncRNAs. Surprisingly, GmNARK appears to play a crucial role in maintaining the stability of miRNAs and circRNAs during nodulation, because loss of function of GmNARK led to a significantly increased number of differentially expressed miRNAs and circRNAs. Notably that the NNR‐circRNAs are associated with thiamine metabolism and terpenoid backbone biosynthesis, and the NNR‐miRNAs were enriched primarily in glycan biosynthesis and carbon fixation. These findings indicate that GmNARK may regulate the balance of energy metabolism during nodulation through ncRNAs.

Despite the low expression levels of these NNR‐lncRNAs in plant cells, they seem to be functionally connected with the NNR‐mRNAs and NNR‐ncRNAs in inoculated plants during nodulation, forming networks at the organ or organismal level to regulate various related processes. Given the role of GmNARK in inhibiting nodulation and promoting plant growth, it is likely that GmNARK regulates the delicate balance between nodulation and plant growth though these NNR‐lncRNA‐mediated regulatory networks. Here, we demonstrated that lnc‐NNR6788 and lnc‐NNR7059 are two important NNR‐lncRNAs that regulate nodulation by targeting specific mRNAs. The former targets the *GmENOD93* gene family cluster to increase the number of root nodules, thereby promoting nodulation. In contrast, the latter modulates nodulation by targeting a beta‐glucosidase gene that is involved in the release of flavonoids in soybeans. These two lnc‐RNAs are regulated by GmNARK, suggesting that early interaction between rhizobia and soybean and symbiotic nodulation are dynamically regulated at the organ and organismal levels. This discovery expands our understanding of the roles of ncRNAs and the GmNARK‐mediated network in nodulation homeostasis.

The ceRNA networks commonly link the functions of lncRNAs with miRNAs and protein‐coding mRNAs.^[^ ^47^, ^50^ ^]^ Our results revealed that some NNR‐lncRNAs, such as lnc‐NNR4481, harbor miR172 response elements, suggesting that they can function as ceRNAs to post transcriptionally regulate downstream gene expression. We showed that lnc‐NNR4481 can reduce the expression of miR172c and that knocking down lnc‐NNR4481 results in altered expression of miR172c and its target gene *GmNNC1*, thus leading to increased nodulation. This finding is consistent with the positive role of miR172c in nodulation.^[^ ^33^, ^34^ ^]^ Thus, lnc‐NNR4481 is an upstream regulator that modulates nodulation through a miR172c‐mediated ceRNA network (Figure 8L). Previously, we demonstrated that GmNARK negatively regulates miR172c through *GmRR11d*‐mediated downregulation of *GmNINa*.^[^ ^22^ ^]^ Here, we showed that GmNARK also suppresses miR172c and nodulation through a lncRNA‐mediated ceRNA mechanism. Notably, we found that lnc‐NNR4481 is tightly connected to miR172k in leaves, suggesting that this lncRNA plays a crucial role in the regulation and coordination of nodulation and plant growth. Further functional characterization of lnc‐NNR4481 and miR172k in nodulation and shoot growth will reveal the role of the lnc‐NNR4481‐miR172c/k modules in regulating the balance between nodulation and plant growth. Further analysis of miR172c and lnc‐NNR4481 expression in the phloem indicated that both ncRNAs are likely transported bidirectionally between shoots and roots. Additionally, GmNARK may coordinate the transport of miR172c and lnc‐NNR4481 to maintain their homeostasis in roots and leaves, thereby regulating various biological processes during soybean nodulation. Further validation of this interesting observation will provide novel insights into the coordinated regulation of soybean nodulation at the organismal level.

## 4 Limitations of the Study

There are several limitations in our study. First, we did not conduct a detailed classification or subsequent functional studies on the various types of ncRNAs (e.g., newly identified miRNAs and possibly circRNAs). Specifically, we encountered certain technical limitations in the identification and characterization of circRNAs, detection of the ncRNAs and mRNAs with low abundance which may be prone to bias and lack sufficient sensitivity or specificity. Second, we did not explore the binding modes of ncRNAs and their target genes or the underlying mechanisms, because this study focused on transcriptomic analysis of ncRNAs at a particular stage of soybean nodulation at the organ and organismal levels. Nevertheless, our findings offer significant insights for future mechanistic studies of ncRNAs in symbiotic nodulation. Third, our understanding of the bidirectional long‐distance transport of lncRNAs in the systemic regulation of nodulation and their relationship with GmNARK still requires further investigation. This can be achieved by using grafting techniques to detect expression changes and the movement of lncRNAs cytologically and genetically. Finally, because of the difficulty of soybean genetic transformation, the role of NR‐ and NNR‐lncRNAs (such as lnc‐NNR4481) in balancing nodulation and growth, which affects yield, has not yet been experimentally validated. We are currently constructing stable transgenic strains to verify the role of these ncRNAs in soybean nodulation. Due to limitations in conditions, our experiment did not include more tissues (such as root tip, stem tip, and nodule), cover more soybean growth stages, or involve more nodulation‐related mutant materials (e.g., *nod49*). Based on these experimental results, we will analyze the role of ncRNA in soybean symbiotic nitrogen fixation from a more comprehensive and specific perspective. These works are ongoing and will continue in our future research.

## 5 Experimental Section

### Plant Materials and Rhizobium Growth Conditions

In the course of this study, the Soybean cultivar Bragg and its GmNARK mutant *nts1007* were employed for a series of experiments encompassing RNA‐seq, gene cloning, expression profiling, and hairy root transformation. Following standardized procedures, the growth of soybean seedlings and *Bradyrhizobium* (*B*.) *diazoefficiens* strain USDA110 was carefully monitored. The seedlings were cultivated in a growth chamber, maintaining a 16 h photoperiod and 8 h dark cycle at 26 °C. For the purpose of RNA‐seq analysis, young seedlings of Bragg and *nts1007*, harvested 4 days after inoculation (DAI), were inoculated with a *B. diazoefficiens* strain USDA110 suspension in distilled water (30 mL, OD600 = 0.08). Following this inoculation, plant tissues including leaves, cotyledons, and roots were collected for both RNA‐seq and qRT‐PCR assessments, both in inoculated and non‐inoculated conditions.

### RNA Quantification and Qualification

The total quantity and integrity of RNAs were evaluated using the RNA Nano 6000 Assay Kit on the Bioanalyzer 2100 system from Agilent Technologies (CA, USA).

### Library Preparation for RNA Sequencing

For the preparation of RNA libraries, 2 µg of total RNA per sample served as the starting material. Adhering to the manufacturer's guidelines, strand‐specific libraries were generated utilizing the NEBNext UltraTM RNA Library Prep Kit for Illumina (NEB, USA), and index codes were appended to each sample for sequence attribution. Ribosomal RNA was eliminated from the total RNA, and the resulting rRNA‐depleted residue was purified through ethanol precipitation. Additionally, for the specific purpose of circRNA sequencing, linear RNA was digested using 3 U of RNase R (Epicentre, USA) per µg of RNA. Fragmentation of the RNA was achieved by utilizing divalent cations under elevated temperatures in the NEBNext First Strand Synthesis Reaction Buffer (5X). Subsequently, random hexamer primers and M‐MLV Reverse Transcriptase (RNase H) were employed for the synthesis of the first‐strand cDNA. For the second‐strand cDNA synthesis, DNA Polymerase I and RNase H were used, with dUTP substituted for dTTP. Exonuclease/polymerase activities were then leveraged to convert any remaining overhangs into blunt ends. Following the adenylation of the 3′ ends of the DNA fragments, the NEBNext Adaptor, featuring a hairpin loop structure, was ligated for hybridization.

To select cDNA fragments preferably ranging from 250 to 300 base pairs (bp) in length, the library fragments were purified using the AMPure XP system (Beckman Coulter, Beverly, USA). Following this, the second strand was digested using USER Enzyme (NEB, USA) at 37 °C for 15 mins, followed by 5 mins at 95 °C, prior to the PCR amplification step. The PCR was conducted using Phusion High‐Fidelity DNA polymerase, Universal PCR primers, and Index (X) Primer. To ensure the quality of the resulting library, it was initially quantified using a Qubit2.0 Fluorometer. The library was then diluted to a concentration of 1.5 ng µL^−1^, and the insert size was verified using the Agilent 2100 bioanalyzer. Once the insert size met the desired specifications, quantitative real‐time PCR (qRT‐PCR) was employed to further assess the library quality.

### Clustering and Sequencing

After satisfactory evaluation of the library quality, libraries are pooled based on their effective concentrations and the intended data output. Subsequently, these pooled libraries are sequenced using the Illumina NovaSeq 6000 platform, generating paired‐end reads of 150 bp each. The sequencing methodology relies on the “Sequencing by Synthesis” principle, which combines synthesis and sequencing in a concurrent process. To initiate sequencing, the sequenced flow cell is supplemented with four fluorescently tagged dNTPs, DNA polymerase, and splice primers. As the sequence clusters extend their complementary strands, each fluorescently labeled dNTP releases its specific fluorescence signal. The sequencer detects these fluorescence signals and, with the aid of computer software, converts the optical signals into sequencing peaks. This process ultimately provides the desired sequence information of the fragments being analyzed.

### RNA Quality Control

The initial fastq formatted raw data underwent rigorous preprocessing via customized Perl scripts, aimed at eliminating reads contaminated with adapters, ploy‐N sequences, or those of poor quality. This step additionally quantified the Q20, Q30, and GC content of the refined dataset. All subsequent analyses relied solely on this high‐quality, cleaned data.

### Reads Mapping to the Reference Genome

The reference genome and its corresponding gene model annotation files were procured directly from the official genome repository. Subsequently, a reference genome index was constructed using Hisat2 v2.0.5, which facilitated the alignment of paired‐end, cleaned reads to the reference genome. Hisat2 was chosen due to its ability to generate a splice junction database based on the gene model annotation, thereby enhancing mapping accuracy compared to other non‐splice‐aware mapping tools.

### Quantification of Gene Expression Levels

Utilizing Stringtie (version 1.3.3b), this work quantified the number of reads mapped to individual genes. Subsequently, we calculated the Fragments Per Kilobase of transcript sequence per Million base pairs sequenced (FPKM), which serves as an expected count. FPKM is a widely employed normalization technique for expression levels, accounting for both sequencing depth and genome size, thus offering a simplified yet robust approach for data standardization, all sequencing work is completed by Novogene Bioinformatics Technology Co., Ltd (Beijing, China).

### Differential Expression Analysis

Prior to conducting differential gene expression analysis, the read counts for each sequenced library were normalized using a scaling factor implemented by the edgeR software package. Differential expression analysis between two samples was subsequently executed utilizing the edgeR R package (version 3.22.5). To account for false positives, the *p*‐values were adjusted employing the Benjamini‐Hochberg method. Genes exhibiting significant differential expression were filtered according to the criterion of an adjusted *p*‐value below 0.05.

### Small RNA Annotation

To maintain the uniqueness of each small RNA's annotation, we adhered to a prioritized sequence: known miRNA, rRNA, tRNA, snRNA, snoRNA repeat, gene, NAT‐siRNA, novel miRNA, and finally ta‐siRNA. In our sample, we obtained an ideal overall rRNA proportion of less than 60%, indicating the high quality of our sequencing.

### CircRNA Identification

To detect and identify the presence of circRNAs, we utilized two software programs: find_circ and CIRI2; and to visually represent these circRNAs, we generated a circos plot using the Circos software. For predicting the Internal Ribosome Entry Site (IRES) scores, we employed the IRESfinder software. Furthermore, to ascertain whether these circRNA sequences exhibited coding potential, we made use of three distinct tools: CPC, CNCI, and PAFM.

### MiRNA Identification and Analysis

First we mapped small RNA tags were employed to search for known miRNAs, utilizing miRBase20.0 as the reference database. Next, the modified software mirdeep2 and srna‐tools‐cli were applied to identify potential miRNAs and visualize their secondary structures.^[^ ^67^ ^]^ Additionally, custom scripts were utilized to obtain the counts of miRNAs and analyze the base bias at the first position of miRNAs with a specific length, as well as at each position of all identified miRNAs.

In order to exclude the influence of tags from protein‐coding genes, repetitive sequences, rRNA, tRNA, snRNA, and snoRNA, the mapping of small RNA tags was conducted against RepeatMasker, the Rfam database, or specific datasets pertaining to the target species itself. To investigate the secondary structure, pinpoint Dicer cleavage sites, and compute the minimum free energy of unannotated small RNA tags, we utilized a combination of the available software tools, miREvo and mirdeep2.^[^ ^67^, ^68^ ^]^

Identify more novel miRNAs using the unique hairpin structure of miRNA precursors. After summarizing all previous comparisons and annotations, some small RNA tags may be aligned with multiple categories. To ensure that each unique small RNA is assigned an annotation, we implemented a priority system as follow: known miRNAs, rRNA, tRNA, snRNA, snoRNA, repeat sequences, protein coding genes, NAT siRNA, followed by protein coding genes (in case of overlap), new miRNAs, and finally ta siRNA.

We investigated the prevalence of miRNA families discovered in our samples across different species. For known miRNAs, we utilized miFam.dat from MirBase (http://www.mirbase.org/ftp.shtml) to identify their families. On the other hand, we submitted for novel miRNA precursors to Rfam (http://rfam.sanger.ac.uk/search/) to search for potential Rfam families.

To quantify the expression levels of miRNAs, we employed the TPM metric, adhering to the criteria outlined by Zhou.^[^ ^69^ ^]^ Specifically, the normalization formula is as follows: Normalized expression = (mapped reads / total reads) * 1 000 000.

### Target Gene Prediction

To forecast the potential target genes of lncRNAs, we initially computed the Pearson correlation coefficient between each lncRNA and its neighboring mRNA, taking their respective abundances (FPKM) into account. Following this, we narrowed down our analysis to those pairs exhibiting a Pearson correlation coefficient exceeding 0.6. For the prediction of miRNA target genes, we employed miRanda to pinpoint potential miRNA target sites. Subsequently, we utilized psRobot_tar within the psRobot framework for plants, or miRanda for animals, to predict the target genes (mRNA, miRNA, and circRNA) of miRNAs.

### The CeRNA Network Prediction

We utilized RNAinter to investigate intricate interactions between mRNA, lncRNA, and miRNA. This comprehensive resource comprised 24 experimentally validated and 14 computationally derived databases, encompassing both miRTarBase and starBase. To ensure reliability, we focused our subsequent analysis on lncRNA‐miRNA and mRNA‐miRNA relationships that exhibited a confidence level of no less than 0.5. In order to uncover competing relationships between mRNA and lncRNA, specifically those sharing common miRNAs during soybean nodulation, we relied on the Pearson correlation test and the hypergeometric test, both of which leveraged the expression profiles. Ultimately, we employed Cytoscape (v3.10.1) software to visualize the intricate co‐expression network, highlighting the overlapping lncRNA‐miRNA‐mRNA relationships.

### The WGCNA Analysis

To preprocess the gene expression values, we preprocessed gene expression values in the form of log_2_(TPM + 0.001) and selecting genes based on their descending variance. The weighted co‐expression relationships between genes were calculated using pairwise Pearson coefficients, resulting in an adjacency matrix. The soft threshold power was determined as the minimal value that achieves a scale‐free topology fit R^2 index exceeding 0.75. After determining this threshold, we transformed the co‐expression matrix into an adjacency matrix using it. The selection of the soft threshold adhered to a standard scale‐free distribution. Scale‐free co‐expression networks were constructed, with a minimum module size of 30 RNAs and a dendrogram cut height of 0.25 for module merging. The application of the soft threshold ensured a scale‐free network structure, where genes with high correlation are grouped into identical modules after the formation of the co‐expression network.

### GO and KEGG Enrichment Analysis

Utilizing the clusterProfiler (3.8.1) R package, we conducted a Gene Ontology (GO) enrichment analysis to identify significantly enriched GO terms among differentially expressed genes. This analysis accounted for gene length bias, and GO terms with corrected *p*‐values below 0.05 were deemed statistically significant. Additionally, we employed the clusterProfiler to assess the statistical enrichment of differentially expressed genes in KEGG pathways(http://www.genome.jp/kegg/).

### SNP, AS, and Fusion Gene Analysis

We used GATK (version 4.1.1.0) software to do the SNP calling procedure, and the resulting raw VCF files underwent rigorous filtering using the GATK's standard filter method and additional parameters including cluster: 3, WindowSize: 35, QD < 2.0, and FS > 30.0. Furthermore, the analysis of alternative splicing, a crucial post‐transcriptional regulatory mechanism, was conducted utilizing the rMATS (version 4.1.0) software. The detection and comprehensive analysis of fusion genes was achieved using the STAR‐Fusion v1.9.0 software. The complete list of software and parameters utilized in this study is detailed in Table S14, Supporting Information.

### Construction and Aesthetic Enhancement of Systematic Evolutionary Trees

In order to study the relationship between homologous genes, phytozome (https://phytozome‐next.jgi.doe.gov/) blast was used to target the homologous genes of the target gene, and the full‐length protein sequences were downloaded. MEGA11 was then employed to analyze and construct the evolutionary tree.^[^ ^70^ ^]^ ITOL was utilized to beautify and group the evolutionary tree for display.^[^ ^71^ ^]^

### Cloning of the Full‐Length LncRNA

Total RNA was extracted and subsequently prepared for the synthesis of both 3′ and 5′ cDNA strands, utilizing an oligo(dT) primer for the 3′ end and a specific primer for the 5′ end, respectively. To clone the 5′ 7‐methylguanylate cap and 3′ polyA tail sequences, a FirstChoice RLM‐RACE kit was employed. Based on the RNA‐seq data of lnc‐NNR4481, primers were specifically designed to amplify both the 5′ and 3′ termini. The resulting amplified fragments were definitively characterized through Sanger sequencing, using Snap Gene (Version 4.1.8) for sequence alignment. The primer sequences used in this study are detailed in Table S15, Supporting Information.

### Plasmid Construction

In order to generate the miR172c overexpression construct, we adhered to the protocol outlined by Wang,^[^ ^73^ ^]^ specifically incorporating a 220 bp fragment of the pre‐miR172c sequence downstream of the 35S promoter in the pEGAD (EV1) vector. Similarly, for the STTM172c construct, we followed the Yan's protocol and inserted an STTM172c fragment beneath the 35S promoter of the pEGAD vector.^[^ ^77^ ^]^ Regarding the lncRNA RNAi assay, we employed a modified version of the pGFP‐930 (EV2) vector,^[^ ^78^ ^]^ which involved replacing the hygromycin gene with a GFP gene derived from the pZHY930_2017 vector. Sequences of lnc‐NNR6788, lnc‐NNR7059 and lnc‐NNR4481 were amplified from a Bragg DNA template, and then cloned. them for hairy root experiments. To validate the interaction between miR172c and the lnc‐NNR4481, we constructed a pEGAD‐GUS (GUS‐EV) vector by swapping the GFP gene with a *GUS* gene. Further, the GUS‐lnc‐NNR4481 construct was created by amplifying the full‐length sequence of lnc‐NNR4481 from a Bragg cDNA template and subsequently inserting it between the GUS gene and the NOS terminator. To generate the GUS‐lnc‐NNR4481‐MU construct, we designed specific primers that encompassed a mutated sequence at the miR172c binding site of lnc‐NNR4481. The resulting lnc‐NNR4481‐MU fragment was then amplified from the lnc‐NNR4481 template and inserted into the pEGAD‐GUS vector, specifically between the *GUS* gene and the NOS terminator. The complete sequences of all the cloned fragments are detailed in Table S15, Supporting Information.

### Soybean Hairy Root Transformation and Rhizobium Inoculation

To validate the role of lncRNAs in soybean nodulation, we performed hairy root transformation. Using *Agrobacterium rhizogenes* K599 harboring the specific construct, composite plant roots were generated, following established protocols.^[^ ^72^ ^]^ Prior to inoculation with *B. diazoefficiens* strain USDA110, the composite plants were irrigated with a nitrogen‐deficient solution, following the guidelines established by Wang.^[^ ^73^ ^]^ After a 10‐day recovery, the plants were inoculated with a freshly prepared suspension of *B. diazoefficiens* strain USDA110 (30 mL, OD600 = 0.08). At 21 DAI, the nodule count per hairy root was recorded. Samples of the roots were then collected for DNA and RNA extraction. The DNA was subsequently used to detect the presence of the selection marker gene via PCR. Meanwhile, the RNA was utilized for qRT‐PCR analysis to identify transgenic hairy roots, which was subsequently followed by an assessment of the nodule count per transgenic root.

### RNA Extraction and Quantitative PCR Analysis

Extraction of total RNA from plant samples was achieved utilizing TRIzol reagent sourced from Aidlab Biotechnologies Co. Ltd. in Beijing, China. For the detection of both mRNA and LncRNA, cDNA strands were synthesized from the total RNA using the Hifair II 1st Strand cDNA Synthesis SuperMix kit from Yeasen Biotech, with *GmELF1b* serving as the internal control. As for miRNA detection, the miRNA cDNA strands were synthesized employing a Stem‐loop‐specific reverse transcription method, as previously described.^[^ ^73^, ^74^ ^]^ Here, miR1520d served as the reference miRNA gene, following the guidelines of Kulcheski.^[^ ^75^ ^]^ For the detection of circRNA, total RNA was first treated with RNAse R from Beyotime Biotechnology for 30 mins at 37 °C, followed by circRNA purification.^[^ ^76^ ^]^ Subsequently, circRNA cDNA strands were synthesized using the Hifair II 1st Strand cDNA Synthesis Kit from Yeasen Biotech, with a random primer, and *Actin7* as the reference gene. To fractionate the nuclear and cytoplasmic components, 8‐day‐old Bragg seedlings were utilized. The Nuclear RNA and cytoplasmic RNA were isolated using the Cytoplasmic & Nuclear RNA Purification Kit from Norgen Biotek. cDNA strands were then synthesized using the Hifair II 1st Strand cDNA Synthesis Kit from Yeasen Biotech, along with gene‐specific primers. In this process, *U6* served as the nuclear marker, while *Actin7* functioned as the cytoplasmic marker. All quantitative PCR analyses were performed utilizing the Hieff qPCR SYBR Green Master Mix kit from Yeasen Biotech, with gene‐specific primers listed in Table S15, Supporting Information.

### Histochemical GUS Staining and Fluorometric of GUS Activity

The GUS‐EV, GUS‐lnc‐NNR4481, and GUS‐lnc‐NNR4481‐MU constructs were co‐transformed into the leaves of 6‐week‐old *N. benthamiana* plants, along with either pEGAD‐EV or *35S::pre‐miR172c*. After 48 h of incubation, the leaves were individually harvested into 50 mL tubes for subsequent histochemical GUS staining and fluorometric assessment of GUS enzyme activity. These assays were performed following established protocols.^[^ ^79^ ^]^ Additionally, leaf samples from each treatment were also collected for qRT‐PCR analysis to quantify gene expression levels.

### Collection of Soybean Phloem Sap

Prepare the cleaning solution: Na_2_EDTA (2‐Na‐ethylenediamine tetraacetic acid, 5 mmol), pH 7.0/KOH; collecting solution: Na_2_EDTA (2.5 mmol), MES [2‐(*N*‐morpholino) ethanesulphonic acid, 1.0 mmol], pH 7.0/KOH.^[^ ^80^ ^]^ A sterile blade was used to sever the above‐ground and below‐ground portions at the stem‐root junction. For the below‐ground portion, the wound was rinsed with detergent solution three times, drying the liquid with sterile blotting paper after each rinse. Subsequently, a RNase‐free pipette was used to aspirate the exuded sap into a clean and sterile tube containing the collection solution. After cutting off the above‐ground portion of the plant, the wound was rinsed with detergent solution twice, blotting dry after each rinse, and placed it into a 50 mL tube filled with detergent solution (Figure S9B, Supporting Information). After a 30‐mins dark treatment, the above‐ground portion was rinsed three times with the collection solution and place it into a new 50 mL tube containing the collection solution. The sap was then collected in the dark for 60 mins. After sample collection was completed, the samples were immersed in liquid nitrogen for at least 30 mins, then transferred to a vacuum freeze‐dryer for processing until only powder remains.

### Statistical Analysis

All analyses were conducted without blinding. We have detailed the screening criteria for RNA seq data in the corresponding sections. The Shapiro‐Wilk test was used to assess the normality of the data. For paired testing, the *t*‐test was employed. For nonparametric datasets involving multiple comparisons, Kruskal‐Wallis one‐way analysis of variance (ANOVA) was utilized, followed by the Holm's Step‐down Bonferroni procedure to adjust the *p*‐values. Comparisons between two groups were conducted using the Mann‐Whitney *U* test. Parametric data were analyzed using one‐way ANOVA, with either Dunnett's post‐hoc test or Tukey's adjustment for multiple comparisons, as detailed in the respective figure captions. Data presentation, statistical significance and sample size for each statistical analysis, were established at a *p*‐value threshold of <0.05. The statistical analyses were performed using GraphPad Prism software (version 9.0.0). Data analysis and visualization were conducted using R packages.

## Conflict of Interest

The authors declare no conflict of interest.

## Author Contributions

Y.L. and C.C. contributed equally to this work. X.L., Y.L., and C.C. designed the experiments. Y.L., C.C., W.C., H.L., and R.X. performed the experiments and analyzed the data. Y.L. did bioinformatic analyses. X.L., Y.L., C.C., and H.J. wrote the manuscript. All authors discussed the results and made comments on the manuscript.

## Supporting information



Supporting Information

Supporting Information

## Data Availability

The data that support the findings of this study are available from the corresponding author upon reasonable request.
