# Supplementary material for: A Comprehensive Transcriptome Atlas Reveals the Crucial Role of LncRNAs in Maintaining Nodulation Homeostasis in Soybean
Source: Adv Sci (Weinh). 2024 Dec 24;12(7):2412104. doi: 10.1002/advs.202412104 (PMC11831499; doi:10.1002/advs.202412104)
Supplement: Supplementary file 1 — Supporting Information [file ADVS-12-2412104-s001.docx]

Supporting Information

A Comprehensive Transcriptome Atlas Reveals the Crucial Role of LncRNAs in Maintaining Nodulation Homeostasis in Soybean

*Yanru Lin§, Chong Chen§, Weizhen Chen, Hangcheng Liu, Renhao Xiao,* *Hongtao Ji and Xia Li**

**SI-1. Table S1. Sample expression information, related to Figure 1 and Figure S1.**

Table S1A. Summary table of sample data quality, related to Figure 1.

Table S1B. Sample correlation, related to Figure S1.

Table S1C. Sample mRNA expression level, related to Figure 1 and Figure S1.

**SI-2. Table S2. NR-mRNA information, related to Figure S1.**

Table S2A. NR-mRNA list, related to Figure S1.

Table S2B. Top expression in three tissues, related to Figure S1.

**SI-3. Table S3. NR-lncRNA expression and function analysis, related to Figure 2.**

Table S3A. NR-lncRNA list, related to Figure 2.

Table S3B. Nodulin-nonregulated lncRNAs, related to Figure 2.

Table S3C. NR-lncRNA coexpression GO Enrichment, related to Figure 2.

**SI-4. Table S4. AS information, related to Figure S2.**

**SI-5. Table S5. NR-circRNA and chromosome distribution information, related to Figure 2 and Figure S2.**

Table S5A. NR-circRNA, related to Figure S2.

Table S5B. mRNA chromosome distribution information, related to Figure 2.

Table S5C. lncRNA chromosome distribution information, related to Figure 2.

Table S5D. circRNA chromosome distribution information, related to Figure 2.

**SI-6. Table S6. NR-miRNA information, related to Figure 3 and Figure S3.**

Table S6A. NR-miRNA, related to Figure 3 and Figure S3.

Table S6B. NR-miRNA target gene, related to Figure 3 and Figure S3.

**SI-7. Table S7. NNR-mRNA and NNR-lncRNA information, related to Figure 4 and Figure S4.**

Table S7A. NNR-mRNA list, related to Figure 4 and Figure S4.

Table S7B. DE_NARK_lncRNA list, related to Figure 4 and Figure S4.

Table S7C. DE_NARK_lncRNA for tissue, related to Figure 4 and Figure S4.

Table S7D. WGCNA-lncRNAs, related to Figure 4 and Figure S4.

Table S7E. NNR-lncRNA, related to Figure 4 and Figure S4.

Table S7F. GO enrich of NNR-lncRNA, related to Figure 4 and Figure S4.

**SI-8. Table S8. NNR-circRNA and NNR-miRNA information, related to Figure S5**.

**SI-9. Table S9.** **mRNA-lncRNA-miRNA network information, related to Figure 5 and Figure S6.**

Table S9A. Target mRNA of NNR-miRNAs and NNR-lncRNAs, related to Figure 5.

Table S9B. lncRNA-miRNA, related to Figure 5.

Table S9C. mRNA-lncRNA-miRNA, related to Figure 5 and Figure S6.

**SI-10. Table S10. Input information for Cytoscape Network, related to Figure S5 and Figure S6.**

**SI-11. Table S11. Gene annotation of mRNAs used in qRT-PCR, related to Figure 6 and Figure S7.**

**SI-12. Table S12.The information about lnc-NNR6788 and lnc-NNR7059, related to Figure 7.**

Table S12A. The expression level of lnc-NNR6788's target genes, related to Figure 7.

Table S12B. Nodule number per hairy root, related to Figure 7.

**SI-13. Table S13. Lnc-NNR4481 related information, related to Figure 8.**

Table S13A. MiR172 family members targeted by lnc-NNR4481, related to Figure 8.

Table S13B. Nodule number per hairy root in empty vector control and RNAi transgenic roots, related to Figure 8

**SI-14. Table S14. The software and parameters used in this study, related to Method.**

**SI-15. Table S15. The primers used in this study, related to Figure 6 ,7 and 8, Figure S6, S7 and S8.**

**SI-16. Figure S1. Nodulation-Regulated mRNAs in all examined tissues, related to Figure 1.**

**SI-17. Figure S2. Nodulation regulation, tissue specificity, and functional annotation of NR-ncRNAs, related to Figure 2.**

**SI-18. Figure S3. Description of NR-miRNAs, related to Figure 3.**

**SI-19. Figure S4. Identification of the GmNARK- and Nodulation-Regulated (NNR) mRNAs and lncRNAs, related to Figure 4.**

**SI-20. Figure S5. Identification of the NNR-circRNAs and the NNR-miRNAs, related to Figure 4.**

**SI-21. Figure S6. The lncRNA-miRNA-mRNA interaction network, related to Figure 5.**

**SI-22. Figure S7. The FPKM and TPM value of selected SNF-related mRNAs, miRNAs, circRNAs, and lncRNAs, related to Figure 6.**

**SI-23.** **Figure S8. The relationship between lnc-NNR6788, lnc-NNR7059 and target genes, related to Figure 7.**

**SI-24. Figure S9** **Lnc-NNR4481 regulates nodulation through the miR172c axis, related to Figure 8.**

**SI-25.** **Sequencing File, related to** **Figure S7D and Figure 8E.**


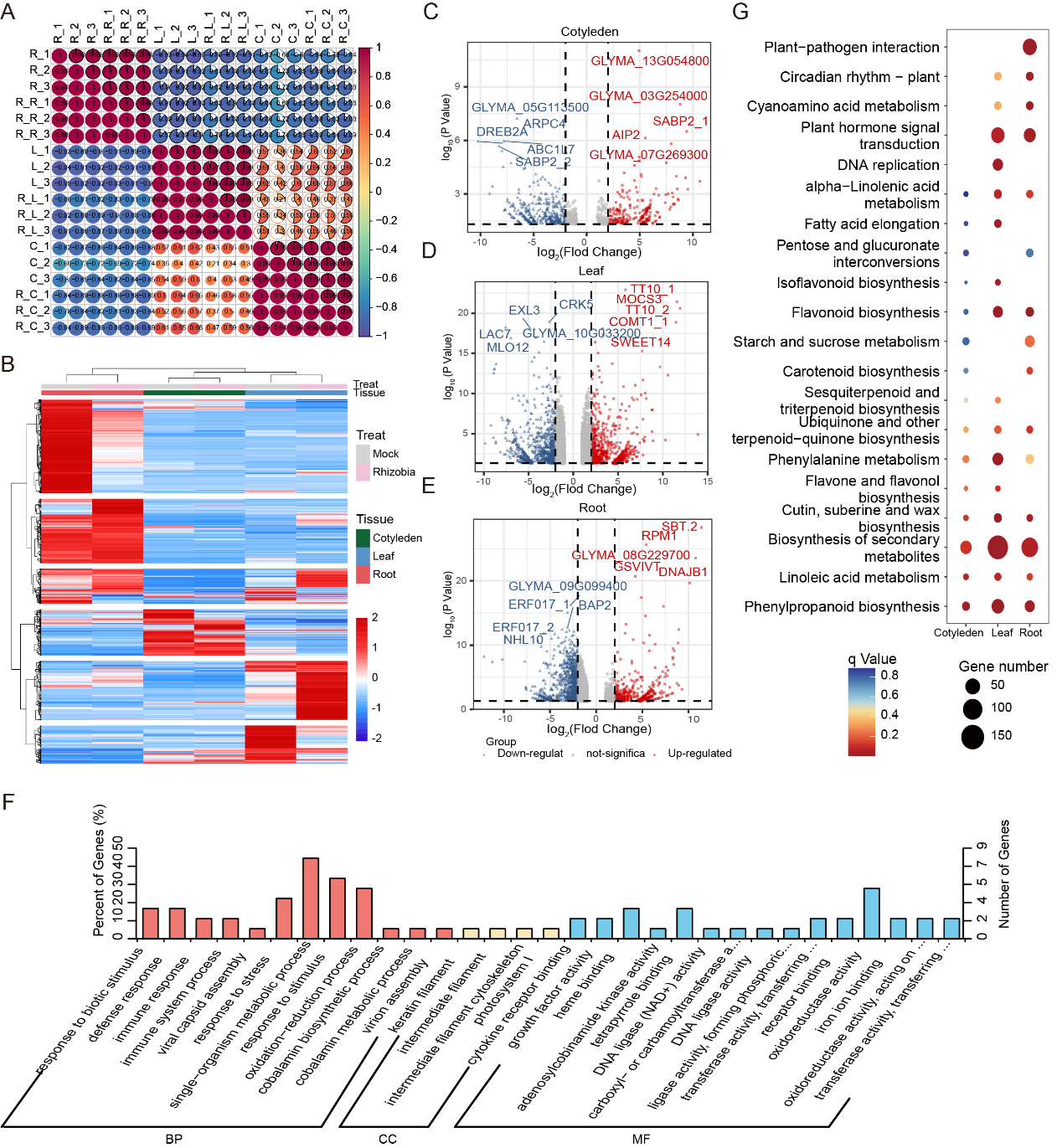


**Figure S1.** Nodulation-Regulated (NR) mRNAs in all examined tissues, related to Figure 1. **A)** Sample correlation coefficient analysis. **B)** The expression pattern of NR-mRNAs during nodulation. **C**-**E)** The volcano plot shows the down-regulation of differentially expressed genes in various tissues, marking the Top 10 differentially expressed genes, (*q* value ≤0.05; log_2_FC ≥ 1). **F)** GO functional analysis of differentially expressed genes in all three tissues. BP, Biological process; CC, Cellular Component; MF, Molecular Function. **G)** Enrichment of KEGG functions in three different tissues.


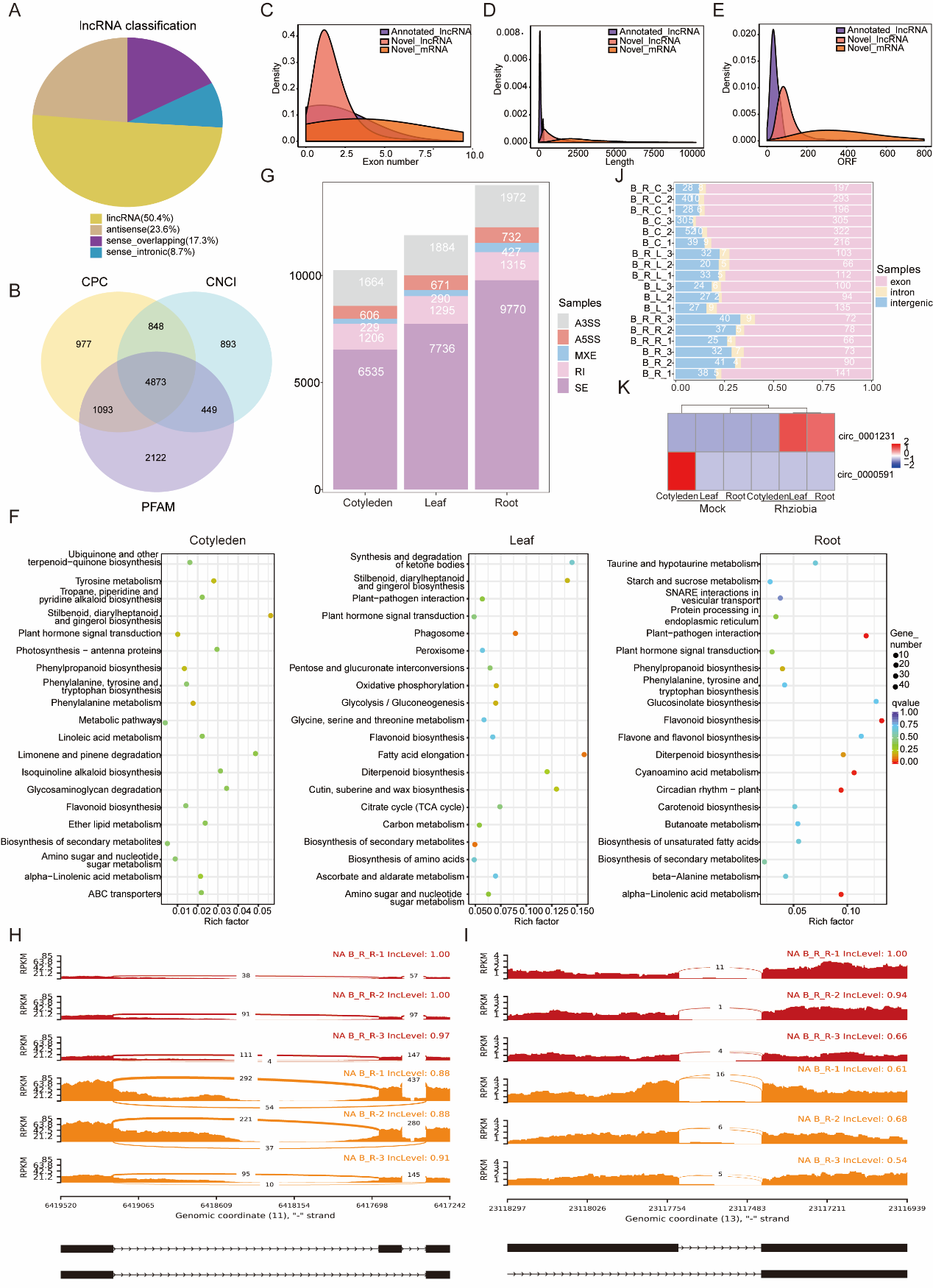


**Figure S2.** Nodulation regulation, tissue specificity, and functional annotation of NR-ncRNAs, related to Figure 2. **A)** LncRNA classification. **B)** Three types of databases are used to identify coding-lncRNAs. **C-E)** Characteristics statistics of lncRNAs. **F)** KEGG functional enrichment of differentially expressed genes in three tissues. **G)** Number of differentially splicing events in each tissue. Different colors represent five types of common AS events. IR: Intron retention, A3SS: Alternative 3′ splicing site, A5SS: Alternative 5′ splicing site, ES: Exon skipping, MXE: Mutually exclusive exons. **H)** Significant different ES in Chr11. **I)** Significant different A3SS in Chr13. **J)** Statistics on the source of circRNAs. **K)** The expression pattern of Nodulation-Regulated circRNAs (NR-circRNA) during nodulation, (*q* value ≤0.05; log_2_FC ≥ 0).


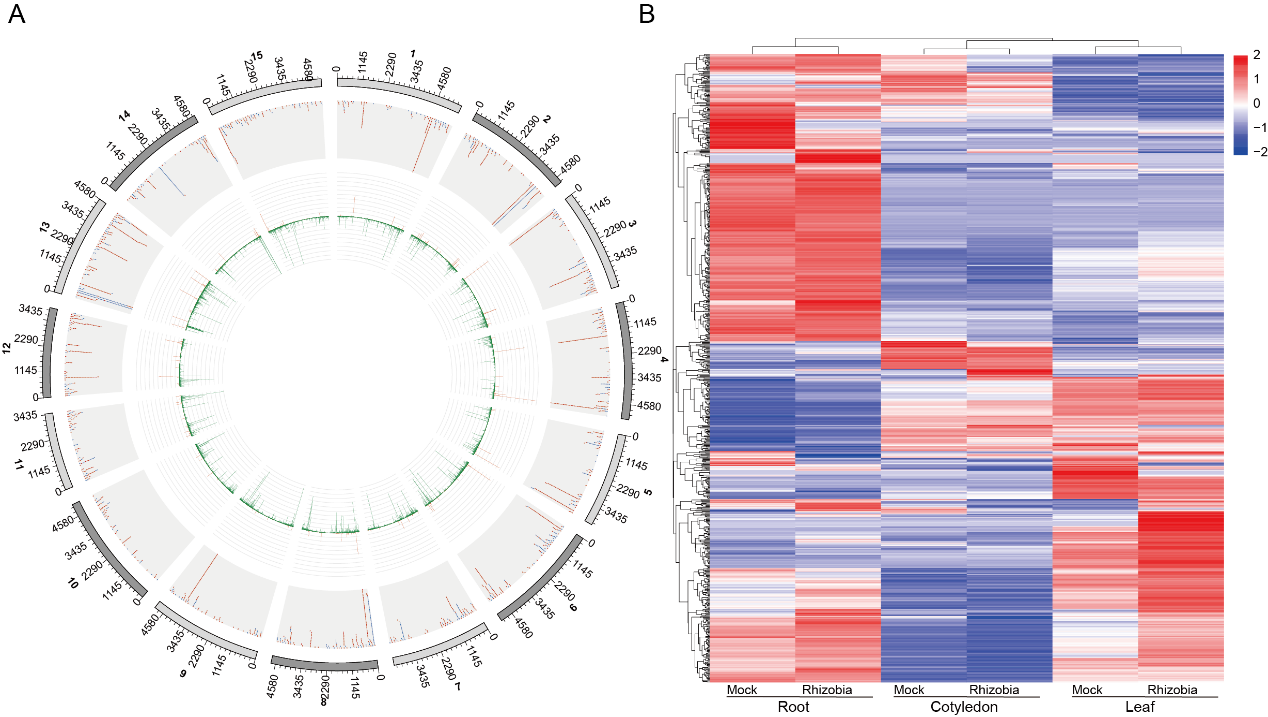


**Figure S3.** Description of NR-miRNAs, related to Figure 3. **A)** Veen shows the chromosomal distribution of NR-miRNAs in various tissues. The outermost circle shows the chromosomes for display; The gray background area in the middle represents the distribution of 10000 reads extracted, with red mapping to positive chains and blue mapping to negative chains; The innermost circle represents all reads aligned to the chromosome, with orange representing the positive chain coverage distribution and green representing the negative chain coverage distribution. Singularities exceeding the mean ± 3 times the standard deviation of all coverage sets were excluded. **B)** The expression pattern of the target genes of NR-miRNAs.


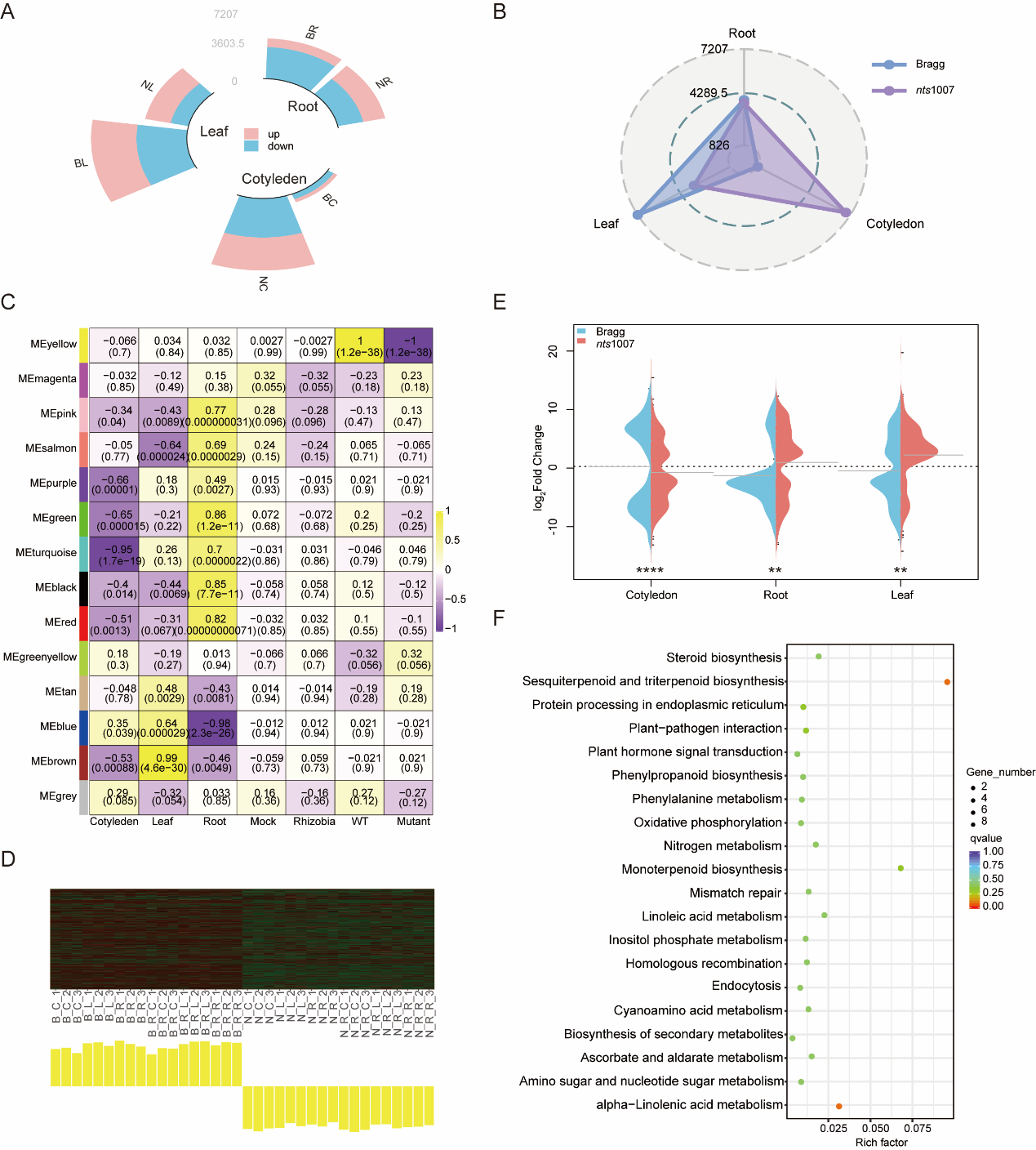


**Figure S4.** Identification of the nodulation*-* and GmNARK-Regulated (NNR) mRNAs and lncRNAs, related to Figure 4. **A)** Number of differentially expressed mRNAs by comparing the Rhizobia infection group to the Mock group. The numbers of up-regulated or down-regulated mRNAs across all tested tissues. B: Bragg; N: *nts1007*. **B)** Number of DE-mRNAs uniquely discovered in different tissues. **C)** WGCNA for all lncRNAs shows the relationship between module and trait. **D)** The expression pattern of lncRNAs in the MEyellow module. **E)** The beanplot shows the effect of inoculation in Bragg and *nts1007* on lncRNA expression levels in three tissues. log_2_Fold Change ≥ 0: up-regulation of expression after rhizobia infection; log_2_Fold Change ≤ 0: down-regulation a rhizobia infection. (student’s *t*-test: ns, not significant; *p < 0.05; **p < 0.01; ***p < 0.001; ****p < 0.0001)**. F)** KEGG functional enrichment of the target genes of NNR-lncRNAs.


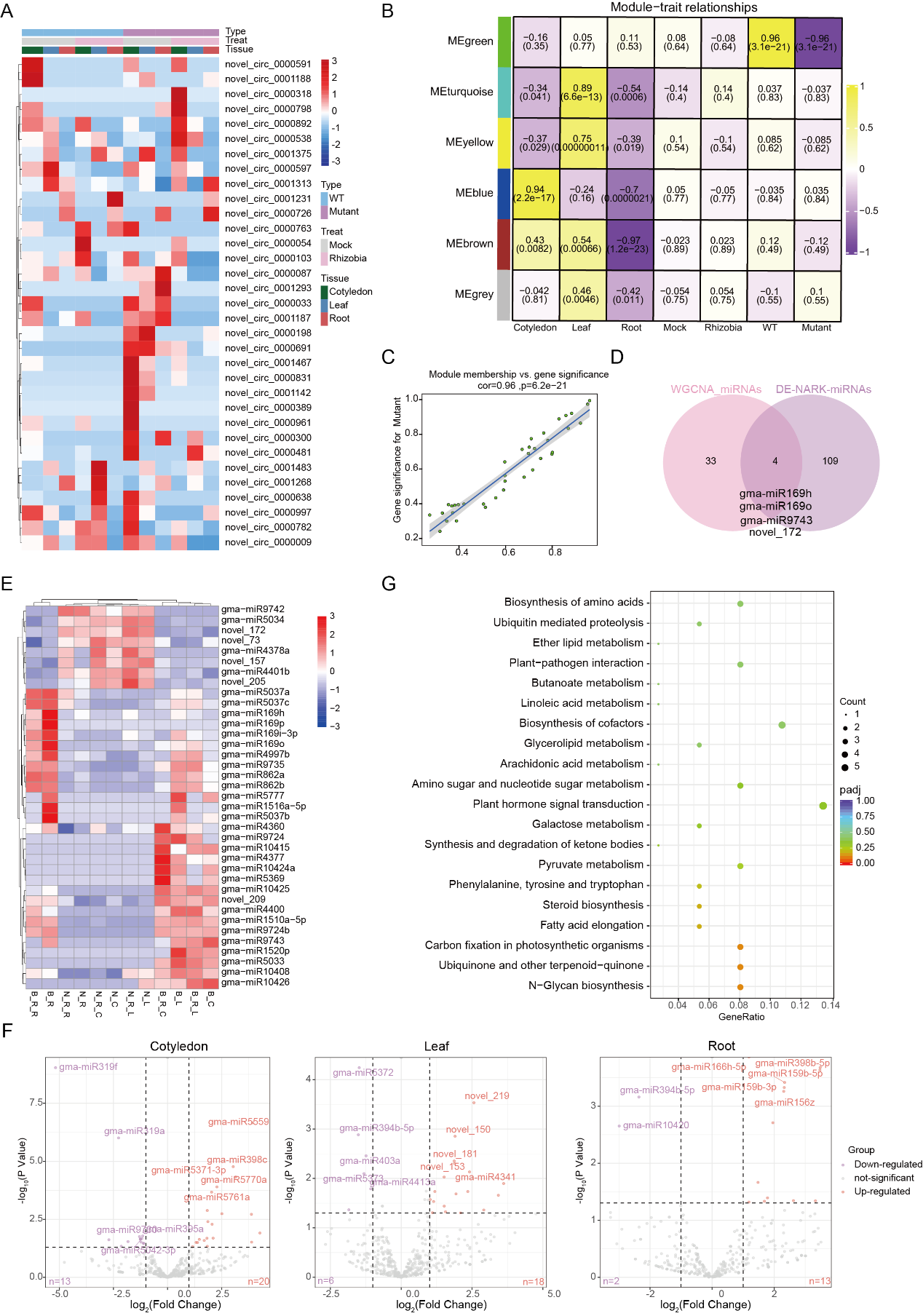


**Figure S5.** Identification of the NNR-circRNAs and the NNR-miRNAs, related to Figure 4. **A)** The expression pattern of NNR-circRNAs. **B)** WGCNA for all miRNAs shows the relationship between module and trait. **C)** The degree of association between genes and traits in the MEgreen module. **D)** A Veen shows the relationship between the differences in miRNAs identified by different methods. **E)** The expression pattern of NNR-miRNAs. **F)** The volcano plot shows the down-regulation of NNR-miRNAs in three tissues, marking the Top 5 differentially expressed miRNAs. **G)** KEGG functional enrichment of the target genes of NNR-miRNAs.

**
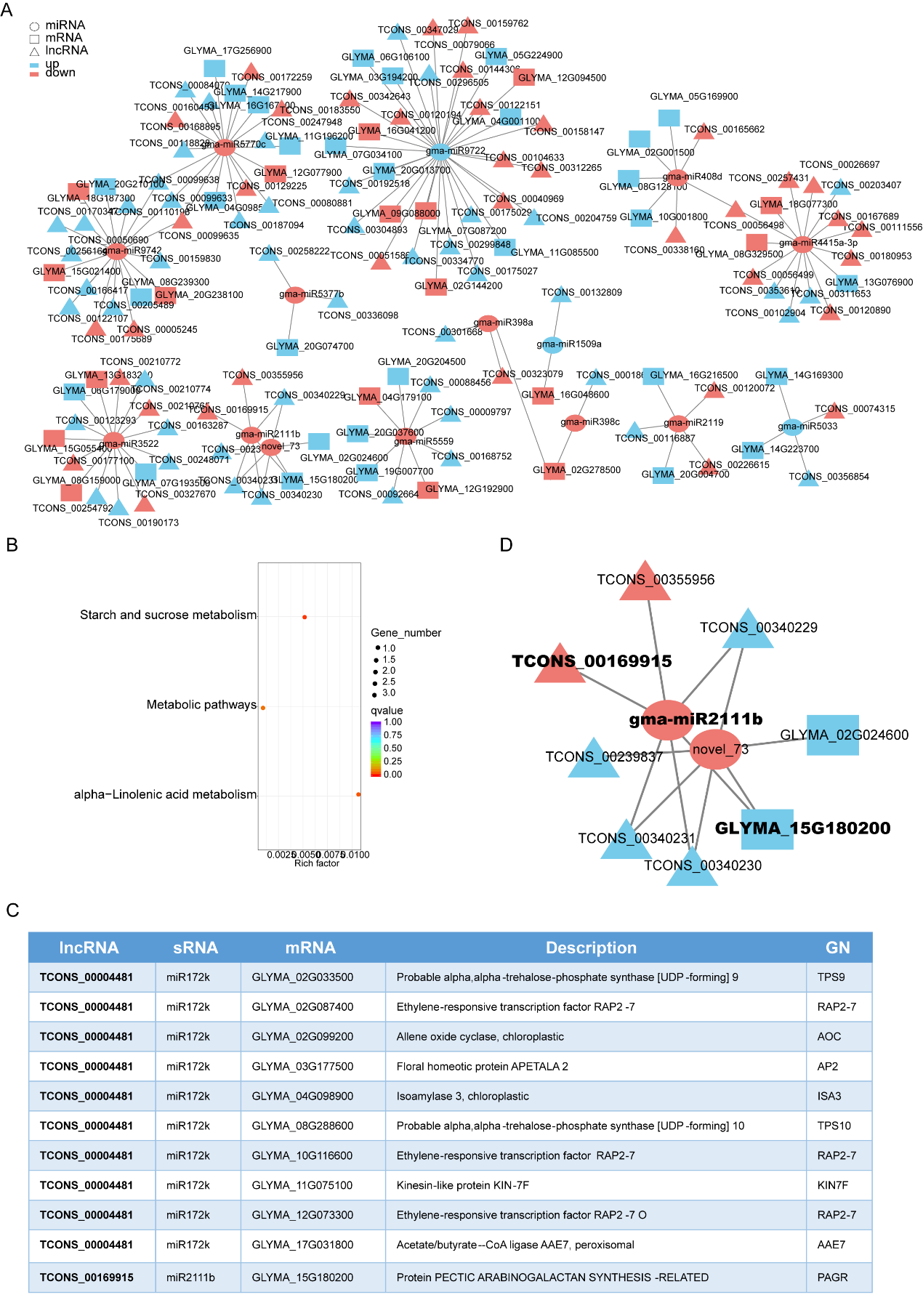
**

**Figure S6.** The lncRNA-miRNA-mRNA interaction network, related to Figure 5**. A)** Part of lncRNA-miRNA-mRNA interaction network diagram. **B)** NNR-lncRNAs targeting miR172k and miR2111b, as well as their target genes. **C)** KEGG rich analysis for the target mRNAs of the lncRNA TCONS0004481 (lnc-NNR4481). **D)** The miR2111b and its predicted target lncRNAs and mRNAs.


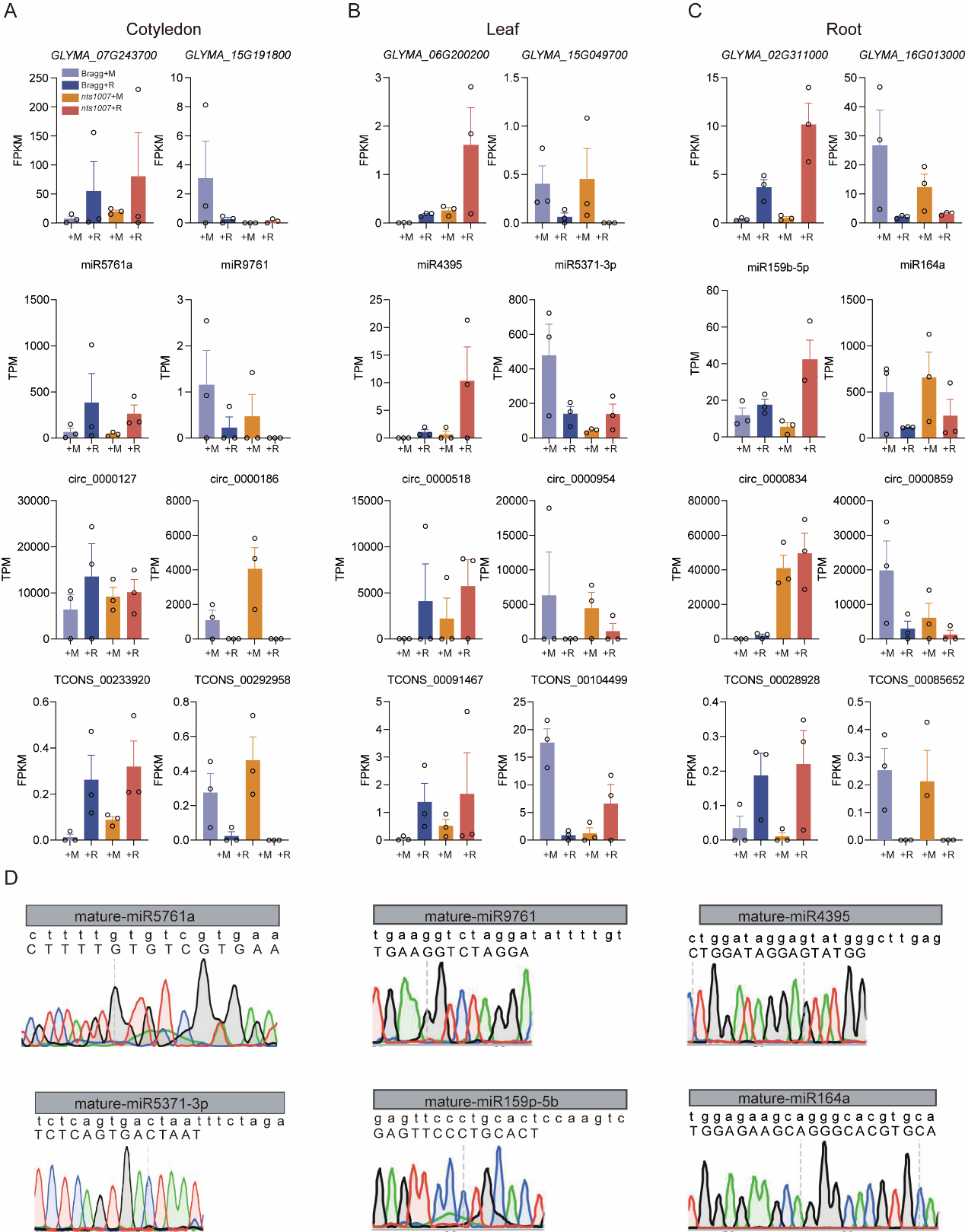


**Figure S7.** The FPKM and TPM value of selected SNF-related mRNAs, miRNAs, circRNAs, and lncRNAs, related to Figure 6**. A-C)** The FPKM and TPM values of some symbiotic nitrogen fixation (SNF) related mRNAs, miRNAs, circRNAs, and lncRNAs in cotyledon **A)**, leaf **B)**, and root **C)**, the expression of which were confirmed by qRT-PCR analysis (*n*=3). Bragg+M: Bragg without inoculation, Bragg+R: Bragg with inoculation, *nts1007*+M: *nts1007* without inoculation, *nts1007*+R: *nts1007* with inoculation. All Data are presented as means ± SEM from the transcriptome data. **D)** Sequencing results for miRNAs (Created by Snap Gene 4.1.8).


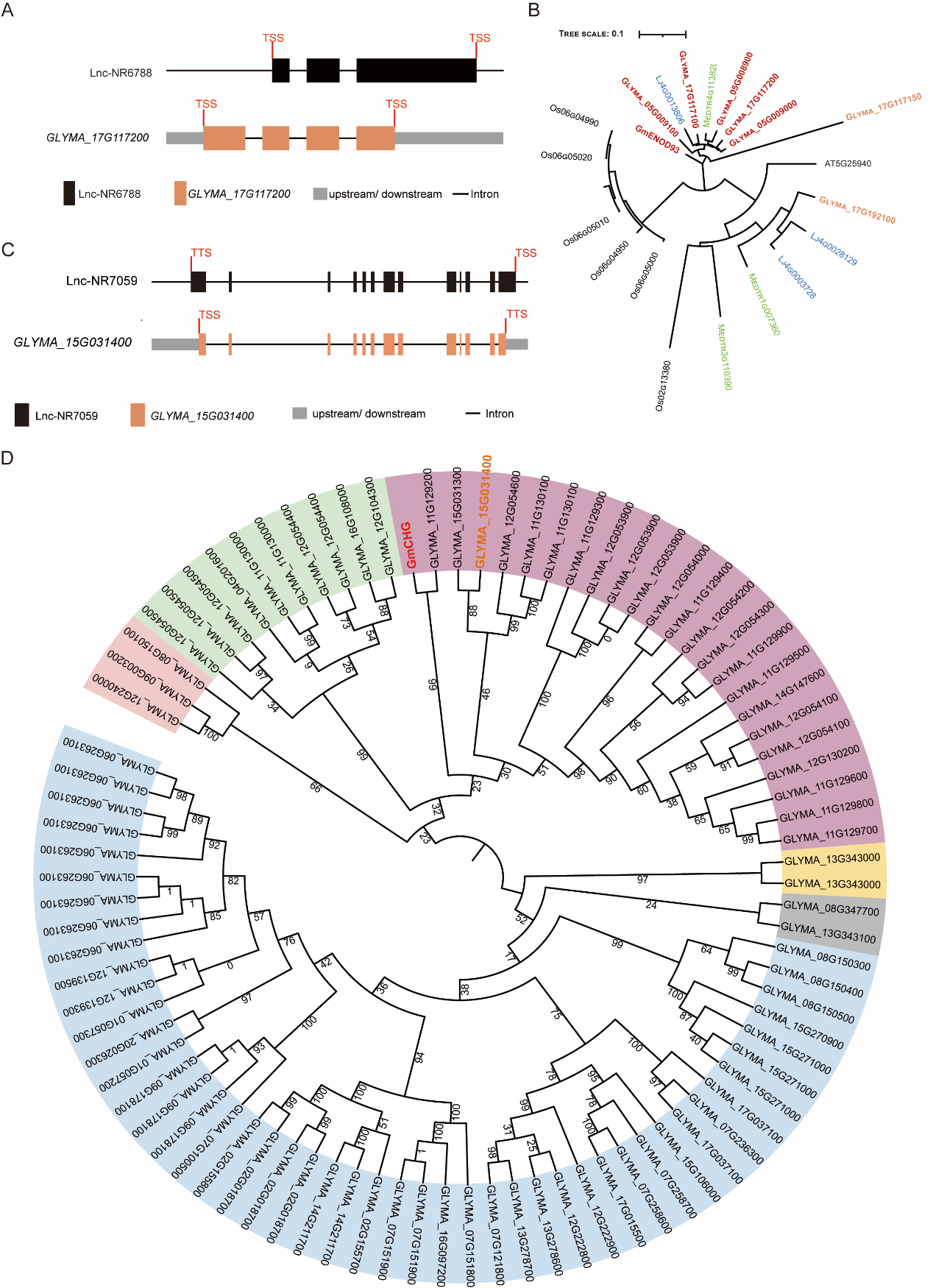


**Figure S8.** The relationship between lnc-NNR6788, lnc-NNR7059 and target genes, related to Figure 7. **A)** Genomic structure of the lnc-NR6788 and *GLYMA_17G117200* locus. Black solid boxes indicate lncRNA exons, yellow boxes indicate gene exons and gray boxes indicate 5’ or 3’ UTR region, red row indicate the direction of transcription. **B)** Orthologs of *GmENOD93*. Using the Neighbor-Joining method, the evolutionary history was deduced, with tree scaled by branch lengths matching evolutionary distances (JTT matrix-based), measured in amino acid substitutions/site. Rate variation across sites was modeled via gamma distribution (shape=1). The study encompassed 22 amino acid sequences, with 219 unambiguous positions after pairwise deletions. Analyses were performed in MEGA11. **C)** Genomic structure of the lnc-NR7059 and *GLYMA_15G031400* locus. Black solid boxes indicate lncRNA exons, yellow boxes indicate gene exons and gray boxes indicate 5’ or 3’ UTR region, red row indicate the direction of transcription. **D)** Paralogs of *GLYMA_15G031400*. Ancestral amino acid states at site 1 were inferred by Maximum Parsimony, with trees initiated by random sequence addition. The study comprised 89 sequences, totaling 966 positions after analysis. Evolutionary analyses were performed in MEGA11.


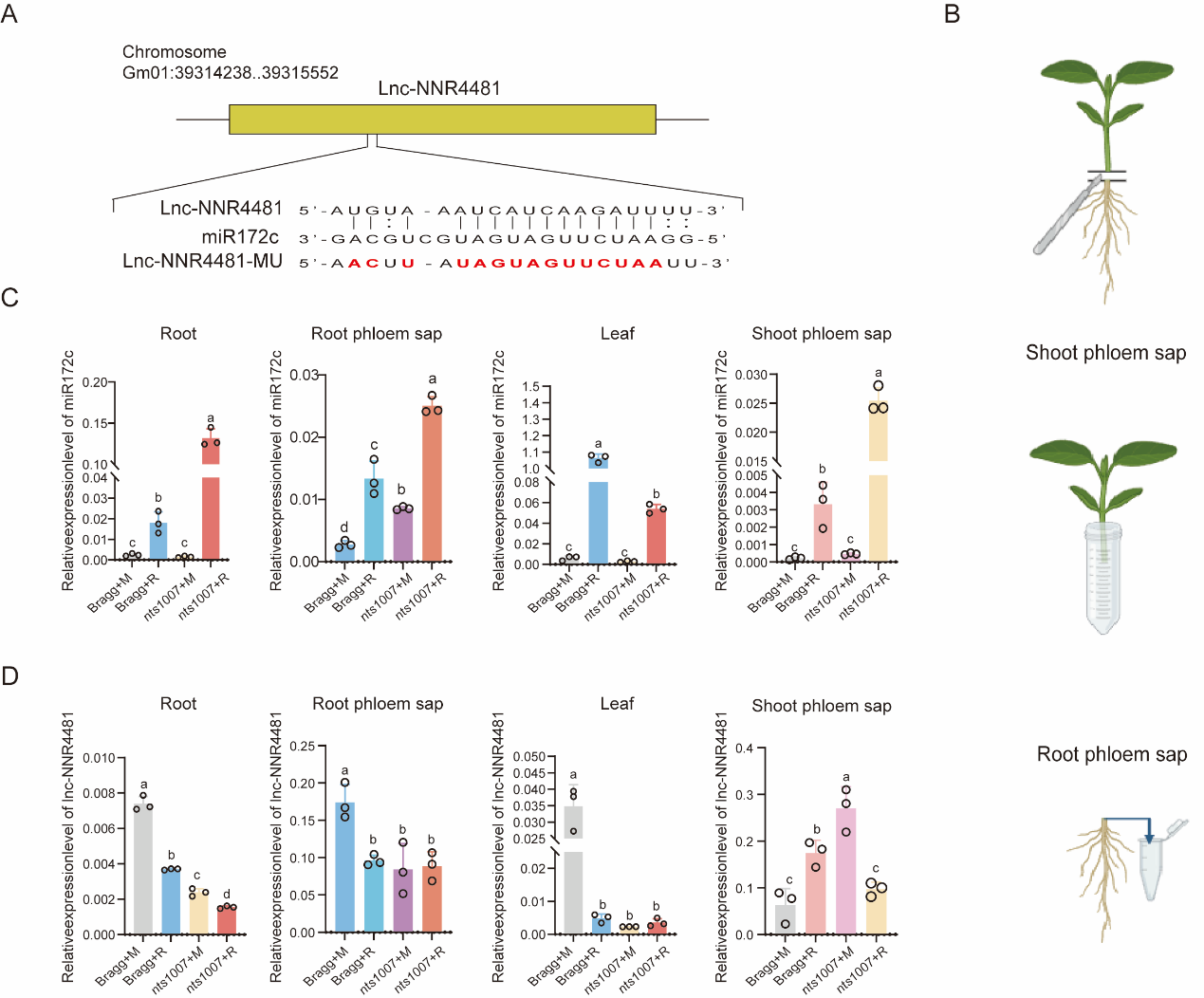


**Figure S9** Lnc-NNR4481 regulates nodulation through the miR172c axis, related to Figure 8. **A)** The complementary region and designed mutations between lnc-NNR4481 and miR172c are depicted. **B)** Schematic diagram of collection location for phloem sap. Cut open the aboveground part of the stem 1-2 centimeters away from the root (black line) and collect the phloem sap flowing out of the plant stem and root (Created in BioRender.com). **C)** The expression levels of miR172c in roots, root phloem sap, leaves and shoot phloem sap exudates (*n*=3). **D)** The expression levels of lnc-NNR4481 in roots, root phloem sap, leaves and shoot phloem sap exudates (*n*=3). Bragg+M: Bragg without inoculation, Bragg+R: Bragg with inoculation, *nts1007*+M: *nts1007* without inoculation, *nts1007*+R: *nts1007* with inoculation (*n*=3). The experiments were repeated for three times. All data are presented as means ± SEM. Different letters indicate significant differences at *p* < 0.05 (One-way ANOVA).
